# Prioritization of Candidate Genes in QTL Regions for Physiological and Biochemical Traits Underlying Drought Response in Barley (*Hordeum vulgare* L.)

**DOI:** 10.3389/fpls.2018.00769

**Published:** 2018-06-12

**Authors:** Kornelia Gudys, Justyna Guzy-Wrobelska, Agnieszka Janiak, Michał A. Dziurka, Agnieszka Ostrowska, Katarzyna Hura, Barbara Jurczyk, Katarzyna Żmuda, Daria Grzybkowska, Joanna Śróbka, Wojciech Urban, Jolanta Biesaga-Koscielniak, Maria Filek, Janusz Koscielniak, Krzysztof Mikołajczak, Piotr Ogrodowicz, Karolina Krystkowiak, Anetta Kuczyńska, Paweł Krajewski, Iwona Szarejko

**Affiliations:** ^1^Department of Genetics, Faculty of Biology and Environmental Protection, University of Silesia, Katowice, Poland; ^2^Department of Botany and Nature Protection, Faculty of Biology and Environmental Protection, University of Silesia, Katowice, Poland; ^3^Department of Developmental Biology, Institute of Plant Physiology, Polish Academy of Sciences, Krakow, Poland; ^4^Department of Plant Physiology, Faculty of Agriculture and Economics, University of Agriculture, Krakow, Poland; ^5^Department of Biotechnology, Institute of Plant Genetics, Polish Academy of Sciences, Poznan, Poland; ^6^Department of Plant Functional Metabolomics, Institute of Bioorganic Chemistry, Polish Academy of Sciences, Poznan, Poland; ^7^Department of Biometry and Bioinformatics, Institute of Plant Genetics, Polish Academy of Sciences, Poznan, Poland

**Keywords:** barley, drought tolerance, function map, QTL, QTL hotspot, CG prioritization, GO enrichment

## Abstract

Drought is one of the most adverse abiotic factors limiting growth and productivity of crops. Among them is barley, ranked fourth cereal worldwide in terms of harvested acreage and production. Plants have evolved various mechanisms to cope with water deficit at different biological levels, but there is an enormous challenge to decipher genes responsible for particular complex phenotypic traits, in order to develop drought tolerant crops. This work presents a comprehensive approach for elucidation of molecular mechanisms of drought tolerance in barley at the seedling stage of development. The study includes mapping of QTLs for physiological and biochemical traits associated with drought tolerance on a high-density function map, projection of QTL confidence intervals on barley physical map, and the retrievement of positional candidate genes (CGs), followed by their prioritization based on Gene Ontology (GO) enrichment analysis. A total of 64 QTLs for 25 physiological and biochemical traits that describe plant water status, photosynthetic efficiency, osmoprotectant and hormone content, as well as antioxidant activity, were positioned on a consensus map, constructed using RIL populations developed from the crosses between European and Syrian genotypes. The map contained a total of 875 SNP, SSR and CGs, spanning 941.86 cM with resolution of 1.1 cM. For the first time, QTLs for ethylene, glucose, sucrose, maltose, raffinose, α-tocopherol, γ-tocotrienol content, and catalase activity, have been mapped in barley. Based on overlapping confidence intervals of QTLs, 11 hotspots were identified that enclosed more than 60% of mapped QTLs. Genetic and physical map integration allowed the identification of 1,101 positional CGs within the confidence intervals of drought response-specific QTLs. Prioritization resulted in the designation of 143 CGs, among them were genes encoding antioxidants, carboxylic acid biosynthesis enzymes, heat shock proteins, small auxin up-regulated RNAs, nitric oxide synthase, ATP sulfurylases, and proteins involved in regulation of flowering time. This global approach may be proposed for identification of new CGs that underlies QTLs responsible for complex traits.

## Introduction

Drought is one of the most devastating abiotic stresses which limits strongly crops growth and productivity. It varies in occurrence, duration and severity, and also from location to location, and in the same location from year to year. Increases in the frequency, severity, and affected areas of droughts are projected due to global climate changes (Baum et al., 2007; Cattivelli et al., 2011; Fang and Xiong, 2015). Plant water deficit occurs when the rate of transpiration exceeds water uptake, and is a component of several different stresses including drought (Bray, 1997). In order to survive and reduce stress damage, plants respond to drought stress with a variety of defense reactions, including stomata closure, limitation of transpiration, repression of photosynthesis and cell growth, activation of respiration, accumulation of osmoprotectants, or antioxidants, although their growth, development, and yield are usually negatively affected (Shinozaki and Yamaguchi-Shinozaki, 2007; Farooq et al., 2009; Cattivelli et al., 2011).

To adapt to water deficit conditions, plants have evolved complex strategies of drought resistance, which integrate multiple adaptations, from the cellular to the whole plant level, and often are specific for particular genotype × environment relationships. Thanks to extensive research, four major drought resistance mechanisms have been described and their indicators dissected: drought escape manifested by a short life cycle, photoperiod sensitivity and plasticity of development; drought avoidance through reduced water loss or increased water uptake; drought tolerance through osmotic adjustment and antioxidant capacity; and drought recovery related to capability to resume the growth after a complete loss of turgor pressure and leaf dehydration (Bray, 2007; Farooq et al., 2009; Chen et al., 2010; Fang and Xiong, 2015). Despite the efforts, much slower progress has been made in understanding genetic basis of drought resistance and in developing more tolerant genotypes, owing to the genetic complexity of this trait and its quantitative inheritance with hundreds of genes of small, often epistatic and/or pleiotropic effects and low heritability (Tuberosa and Salvi, 2007; Cattivelli et al., 2011; Fan et al., 2015).

Barley (*Hordeum vulgare* L.) is the fourth most widely cultivated cereal in terms of harvested acreage and production (FAO, 2016). It is an excellent model plant to decipher genetic background of drought resistance as it has considerable genetic adaptability to a wide range of environments and a high level of drought tolerance showed by local landraces and wild barley (*H. vulgare* spp. *spontaneum)*. Being a valuable resource of alleles for adaptive traits, drought tolerant genotypes have been increasingly exploited in quantitative trait loci (QTL) studies to uncover the genetic control of multiple adaptations to drought stress (Baum et al., 2007; Cattivelli et al., 2011). Over last two decades numerous QTLs controlling agronomic performance and yield components under drought stress have been identified for barley (Teulat et al., 2001b; Baum et al., 2003; Talamé et al., 2004; von Korff et al., 2008; Cuesta-Marcos et al., 2009; Kalladan et al., 2013; Mansour et al., 2014; Tondelli et al., 2014). While these studies were oriented to decipher genomic regions important for barley breeding in order to maintain crop yield and grain quality under drought stress, they have seldom been used to elucidate the mechanisms of drought resistance at the genetic and molecular level (Baum et al., 2007; Fang and Xiong, 2015).

To fulfill this purpose, various indicator traits of physiological/biochemical processes occurring under drought have been investigated in barley using diverse mapping populations and drought stress conditions. Most studies concentrated on the parameters related to plant water status, resulting primarily in the identification of QTLs for relative water content (RWC), and then, for osmotic adjustment (OA), osmotic potential (OP), water content (WC), or carbon isotope discrimination (Teulat et al., 1997, 1998, 2001a, 2003; Diab et al., 2004; Chen et al., 2010; Szira et al., 2011; Wójcik-Jagła et al., 2013; Honsdorf et al., 2014; Fan et al., 2015; Mora et al., 2016). In contrast, only individual studies have detected QTLs for water-soluble carbohydrates (WSC, Teulat et al., 2001a; Diab et al., 2004) and proline content (Sayed et al., 2012; Fan et al., 2015). Additionally, some studies on the photosynthetic efficiency under drought stress conditions have identified QTLs related to chlorophyll fluorescence parameters, chlorophyll content or PSII (photosystem II) photochemical activity (Guo et al., 2008; von Korff et al., 2008; Wójcik-Jagła et al., 2013; Honsdorf et al., 2014; Mora et al., 2016) and plasma membrane integrity (Wójcik-Jagła et al., 2013). Recently, QTLs for multiple metabolites accumulation under drought stress (Piasecka et al., 2017), including QTLs for the content of fat-soluble antioxidants: α- and γ-tocopherols (Templer et al., 2017), have been detected.

These papers present a valuable source of data on the chromosomal regions potentially involved in drought response in barley, however, the comprehensive integration of the majority of these results and the anchoring of the identified QTLs on barley genome to decipher genes and networks for QTLs underlying particular physiological traits could be challenging. The difficulties arise not only from inherent limitations associated with particular mapping populations and applied methods but foremost from a lack of the information on the proper genomic localization of these QTLs which preclude their precise positioning on physical maps (Mir et al., 2012; Salvi and Tuberosa, 2015). Today, more comprehensive and effective approach to decipher the genetic basis of QTLs (Monclus et al., 2012; Bargsten et al., 2014; Kumar et al., 2017) may be applied also in barley. The availability of SNP (single nucleotide polymorphism) markers based on within-gene polymorphisms (Close et al., 2009) together with high-throughput genotyping methods enable production of function maps with high density genome coverage for QTL mapping (Potokina et al., 2008; Mammadov et al., 2012). Such maps provide straight-forward connection of the identified QTL intervals to the reference barley genome sequence (Mayer et al., 2012), which could be followed by the analysis of large candidate gene assemblies and their biological interpretation using public databases (e.g., Gene Ontology; GO) and bioinformatics high-throughput enrichment tools.

This study present a first comprehensive approach for elucidation of genetic basis of physiological mechanisms of drought response/tolerance in barley based on the identification of the positional candidate gene (CG) assembly within the QTL confidence intervals, followed by the exploration of their putative functions related to drought tolerance. The study aimed particularly (1) to identify QTLs for a wide range of physiological/biochemical traits representative for plant water status, photosynthetic efficiency, osmoprotectant and hormone content, as well as activity or accumulation of antioxidants under drought stress on a created high-density function map; (2) to project QTL confidence intervals on physical barley genome map; and (3) to retrieve a set of potentially causative CGs, underlying the analyzed traits using a Gene Ontology (GO) enrichment approach.

## Materials and methods

### Plant material

Barley (*H. vulgare* L.) population of 100 recombinant inbred lines (RIL), named MCam, produced by the single-seed descent (SSD) method from the cross between spring genotypes “Maresi” and Cam/B1/CI08887//CI05761 (here after referred as CamB), was used in this study to phenotype a set of physiological and biochemical traits under drought stress and control conditions, followed by their QTL mapping on a high-density function map. “Maresi” is a German advanced cultivar (semidwarf, two-rowed, malting type), of high and stable yielding under the European environmental conditions (pedigree and seed source: http://genbank.vurv.cz/genetic/resources/asp2/default_a.htm), whereas CamB is a Syrian breeding line (two-rowed, early heading), kindly obtained from ICARDA (International Center for Agricultural Research in the Dry Areas), adapted to dry environments (detailed characteristics of parental genotypes: Górny, 2001; Filek et al., 2015, 2016; Chmielewska et al., 2016).

### Plant growth conditions and drought stress treatment

The experiment was carried out in a phytotron (growth chamber). Seeds of each RIL were sterilized and sown separately in a box (22 L; 10 RIL per day), filled with a mixture of sandy loam and sand (7/2, v/v). In this substrate, a pF range of 2.2–3.0 indicated easily available water whereas pF > 4.2 was the permanent wilting point, as calculated based on the water retention curve (Filek et al., 2015). The pF-value is defined as a logarithm of the pressure *p* (expressed in centimeters of water head) necessary for removal of water from soil capillaries (Mikołajczak et al., 2017). Soil moisture was regulated two times a day. After germination (4 days, at 25°C), the boxes containing 30 plants were kept at 5°C (day/night) during the next 10 days, then the following parameters were settled in phytotron growth chamber: photoperiod 16/8 h (day/night), the temperature of 20/17°C (day/night), irradiance of 520 μmol(photon)m^−2^s^−1^. After 10 days, the number of plants in a box was reduced to 25. The substrate humidity was determined by monitoring boxes weight, and it was kept at 11% water content (VWC), i.e., pF = 2.8. After 13 days (at the moment of the emergence of the 4th leaf) the temperature was set to 25/16°C and soil drought (3.65% VWC, i.e., pF = 4.0) was applied to plants for 10 days. Plants grown in boxes with 11% VWC were used as the control (optimal condition growth). The boxes were randomly distributed in the growth chamber, and their location was changed twice a day. Measurements of the physiological/biochemical traits were performed at the seedling stage, on the third leaf for the plants from both (control and drought stress) conditions after 10 days of drought. Depending on the trait, the measurements were performed in 4–25 biological replications.

### Phenotyping

Altogether, 40 different physiological/biochemical traits were measured at the seedling stage for RILs and the parents (described below and in Table 1). To facilitate the analysis, the traits were grouped into four categories according to their roles in drought stress response: plant water status (5 traits), photosynthetic efficiency (19 traits), osmoprotectant and hormone content (7 traits), activity and accumulation of antioxidants (9 traits).

**Table 1:** Physiological and biochemical traits used in QTL analysis.

**Trait category**	**Abbrev**.	**Physiological/biochemical trait**	**Units**
Plant water status	WC	Water content	g H_2_O/g DW
	WL	Water loss rate	g H_2_O/g DW
	RWC	Relative water content	%
	WUE	Water use efficiency	μmol CO_2_/mmol H_2_O
	EL	Electrolyte leakage	%%
Photosynthetic efficiency	ABS/RC	Light absorption flux (ABS) per PSII reaction center (RC)	%
	TR_0_/RC	Trapped energy flux per PSII reaction center (RC)	%
	ET_0_/RC	Electron transport flux per PSII reaction center (RC)	%
	DI_0_/RC	Dissipated energy flux per PSII reaction center (RC)	%
	ABS/CS	Light absorption flux per excited leaf cross-section at *t* = 0 (CS)	%
	TR_0_/CS	Trapped energy flux per excited leaf cross-section at *t* = 0 (CS)	%
	ET_0_/CS	Electron transport flux per excited leaf cross-section at *t* = 0 (CS)	%
	DI_0_/CS	Dissipated energy flux per excited leaf cross-section at *t* = 0 (CS)	%
	RC/CS	The maximum number of active reaction center (RC) per excited leaf cross-section at *t* = 0 (CS)	%
	ϕ_po_	Maximal quantum yield of primary photochemistry (TR_0_/ABS)	%%
	ψ_o_	Exciton transfer efficiency to the electron transport chain (ET_0_/TR_0_)	%%
	ϕ_eo_	Electron transport yield (ET_0_/ABS)	%%
	(1-B)av	The average fraction of open RC during the time needed to complete the closure of all RCs	%
	PI_abs_	The performance index per absorption	%
	${\text{F}}_{\text{v}}^{\prime }/{\text{F}}_{\text{m}}^{\prime }$	Efficiency of excitation energy capture by open PSII RC	%%
	qP	Photochemical quenching	%%
	Φ_PSII_	Photochemical quantum yield of PSII	%%
	Pn	Net photosynthesis rate	μmol CO_2_/m^2^s
	E	Transpiration rate	mmol H_2_O/m^2^s
Osmoprotectant and hormone content	Pro	Free proline content	μg/g FW
	Glu	Glucose content	μg/mg DW
	Fru	Fructose content	μg/mg DW
	Suc	Sucrose content	μg/mg DW
	Raf	Raffinose content	μg/mg DW
	Mal	Maltose content	μg/mg DW
	Eth	Ethylene content	nl/g FW
Activity and accumulation of antioxidants	SOD	Superoxide dismutase activity	U
	CAT	Catalase activity	U
	POX	Peroxidase activity	U
	GTt	γ-tocotrienol content	μg/mg DW
	ATt	α-tocotrienol content	μg/mg DW
	DTf	δ-tocopherol content	μg/mg DW
	GTf	γ-tocopherol content	μg/mg DW
	ATf	α-tocopherol content	μg/mg DW
	BC	β-carotene content	μg/mg DW

*DW, dry weight; FW, fresh weight; U, arbitrary unit of enzyme activity per mg of total soluble protein*.

#### Plant water status

Relative water content (RWC), water content (WC), water loss (WL) rate, water use efficiency (WUE), and electrolyte leakage (EL) were determined in this category. RWC was calculated according to Barrs (1968), where RWC = (FW – DW)/(TW – DW) × 100%, and where FW, DW and TW, respectively, are fresh, dry and turgid weight. To measure TW, leaf samples were placed in water (7°C) in darkness for 24 h, for the complete rehydration. WC was calculated as (DW/FW) × 100%. WL rate was calculated on the basis of the depletion of leaf FW during 24 h under the appropriate growing conditions. WUE was calculated as Pn/E, where Pn and E are net photosynthesis and transpiration rates, respectively, estimated by the measurement of gas exchange (next section). EL test was used to determine the plasma membrane integrity. For each genotype, samples were prepared as described by Płazek et al. (2014).

#### Photosynthetic efficiency

Photochemical efficiency was estimated by means of chlorophyll *a* fluorescence registration. Measurements were taken using a fast chlorophyll fluorescence induction kinetics fluorometer Handy PEA and modulated fluorescence system FMS2 (Hansatech, Kings Lynn, UK). The induction of a chlorophyll fluorescence signal was measured after 30 min of leaf dark adaptation in clips (Hansatech). Before measurements, the LED-light source of the fluorometer was calibrated using an SQS light meter (Hansatech, Kings Lynn, UK). The conditions used to determine polyphasic chlorophyll *a* fluorescence transients were: excitation irradiance of 3,000 μmol m^−2^s^−1^, a pulse duration of 1 s, and fixed gain of 0.7 (Handy PEA ver. 1.3 software, Hansatech). Fourteen parameters were calculated (Kalaji et al., 2011): ABS/RC and ABS/CS—light absorption flux, respectively, per PSII reaction center (RC) and per excited leaf cross-section at *t* = 0 (CS); ET_0_/RC and ET_0_/CS (electron transport flux per RC and CS); TR_0_/RC and TR_0_/CS (trapped energy flux per RC and CS); DI_0_/RC and DI_0_/CS (dissipated energy flux per RC and CS); RC/CS (the maximum number of active RC per CS); ϕ_po_ (TR_0_/ABS—maximal quantum yield of primary photochemistry); ψ_o_ (ET_0_/TR_0_—exciton transfer efficiency to the electron transport chain); ϕ_eo_ (ET_0_/ABS—electron transport yield); PI_abs_ (the performance index per absorption); (1-B)av (the average fraction of open RC during the time needed to complete the closure of all RCs). FMS2 measurements were made after light adaptation of the leaf (usually 2–5 min at 500 μmol (quanta) m^−2^s^−1^) when the fluorescence signal (Fs) became constant. Three parameters: the photochemical quantum yield of PSII (Φ_PSII_), the photochemical quenching (qP) and the efficiency of excitation energy capture by open PSII RC (${\text{F}}_{\text{v}}^{\prime }/{\text{F}}_{\text{m}}^{\prime }$) were calculated according to Genty et al. (1989). The gas exchange rate (Pn; E) was measured using an infrared gas analyzer (Ciras-1, PP Systems, Hitchin, UK) and Parkinson leaf chamber (PLC6). The controlled measuring conditions were: CO_2_ concentration of 400 μmol (CO_2_) mol^−1^ (air), 30% relative humidity, irradiance of 800 μmol (quanta) m^−2^s^−1^ and the leaf temperature of 25°C. The measurements of chlorophyll fluorescence and gas exchange were carried out from 10 a.m. to 2 p.m. (beginning of day 6 a.m., day length 16 h), and until stable measurement values were obtained. The order of plants measurements (photosynthesis and fluorescence) during each day of was random.

#### Osmoprotectant and hormone content

Seven compounds were quantified in this category. Soluble carbohydrates (glucose, fructose, sucrose, raffinose, and maltose) were identified by their retention times and quantified by integrating peak areas against the internal standard according to the procedure by Janeczko et al. (2010). Measurements were made using a high performance liquid chromatography consisting of the following modules: a gradient pump (Agilent 1200, Santa Clara, CA, USA), autosampler (Agilent 1200), the thermostat STH 585 (Dionex, Sunnyvale, CA, USA), ESA detector Coulochem II Analitical Cell 5040 with a gold working electrode and a palladium reference electrode, an analog/digital converter (Agilent), program control and data collector software ChemStation Rev.B.04.01 (Agilent).

The content of free proline was determined spectrophotometrically (at λ = 520 nm) according to the methods by Bates et al. (1973) and Marin et al. (2009), with the use of UV/VIS spectrophotometer (UV-1800, Rayleigh, Beijing, China) and it was calculated from the standard curve.

For ethylene production, each sample (0.1 g FW) was homogenized in 70% ethanol. Following the centrifugation, the supernatant was evaporated (at 40°C) and the pellet was dissolved in H_2_O (1 ml), and 1 pmol HgCl_2_ was added. Then, the sample was transferred into a new vial and incubated on ice (for 5 min) with a mixture of 5% NaOCl and saturated NaOH (2/1, v/v). The gas sample was taken from the vial, and ethylene was quantified by gas chromatography (Hewlett Packard 5890 Series II, Palo Alto, CA, USA) with Porapak R column (80/100 mesh, Agilent, Santa Clara, CA, USA) and detector (flame ionization) as described by Grzesiak et al. (2013).

#### Activity and accumulation of antioxidants

Antioxidant enzymes (3), fat-soluble antioxidants (5) and β-carotene were quantified in this category. To measure the activity of antioxidant enzymes: superoxide dismutase (SOD), catalase (CAT), and peroxidase (POD), the plant material was homogenized at 4°C in 0.05 mM potassium phosphate buffer (KP) with 0.1 mM EDTA. The supernatant was divided into three subsamples after centrifugation. The activity of SOD [EC 1.15.1.1.] was determined spectrophotometrically by the cytochrome reduction method (McCord and Fridovich, 1969) with the modifications by Szechyńska-Hebda et al. (2012). Changes in absorbance were followed with a Biochrom Ultrospec II spectrophotometer (LKB, Sweden) at λ = 550 nm. The inhibition of oxidized cytochrome c absorbance was monitored. The activity of CAT [EC 1.11.1.6] was measured spectrophotometrically according to Aebi (1984) with the modifications by Wojtania et al. (2016). The decrease in H_2_O_2_ absorbance was measured at λ = 240 nm. The activity of POD [EC 1.11.1.11] was measured spectrophotometrically using the method by Lück (1962) with the modifications by Wojtania et al. (2016). The increase in oxidized p-phenylenediamine absorbance was monitored at λ = 485 nm.

Tocochromanols (α- γ- δ-tocopherols and α- γ-tocotrienols) and β-carotene were measured based on the modified method by Surówka et al. (2016). Briefly, lyophilized samples were extracted in 0.1% solution of butylated hydroxytoluene (BHT) in ethanol/acetone/methanol/2-propanol (8/3/3/1 v/v) at 70°C in shaking water bath for 15 min, then 80% KOH was added, and the extraction was continued for 30 min. Next, samples were diluted with H_2_O (1/1 v/v) and cleaned on Chromabond XTR cartridge (Macherey-Nagel, Germany). Compounds of interest were eluted by n-hexane, vacuum evaporated (Rota Vapor, Switzerland) and reconstituted in 1% BHT in methanol/dichloromethane (3/1 v/v) prior HPLC separation. The Agilent 1260 (Santa Clara, CA, USA) UHPLC binary system with diode array (DAD) and fluorescence (FLD) detectors was used. Separation was achieved on Ascentis Express C-18 (Supelco Analytical, Sigma Aldrich, USA) analytical columns at 0.8 ml/min, 60°C and linear gradient of A) 0.5% formic acid (FA) in acetonitrile (ACN)/H_2_O (6/4 v/v) and B) 0.5% FA in 2-propanol/ACN (9/1 v/v), from 40 to 100% of B in 15 min. Tocochromanols were detected by FLD, whereas β-carotene by DAD. Identity and quantity of compounds were confirmed by the comparison with data obtained for the pure standards under identical conditions.

### Statistical analysis

All physiological traits data were analyzed with Statistica 12.0 software (Stat. Soft Inc., USA). The normality of trait distributions was verified using the Shapiro–Wilk test. Linear correlation coefficients (Pearson's) were calculated between all the analyzed traits separately for drought and control conditions. The *F*-test was used to assess the homogeneity of variance and the Student's *t*-test to compare the statistical significance of differences of the analyzed traits between control and drought conditions.

In the QTL analysis absolute values of all physiological/biochemical traits were used which described the drought response/tolerance level of RILs, and drought stress indices (DSI), which are relative values, were also calculated for all measured traits to compare among RILs (Bouslama and Schapaugh, 1984; Wójcik-Jagła et al., 2013) according to the formula: DSI (%) = (*d/c*) × 100%, where *d* and *c* are the absolute values obtained under drought and control conditions, respectively. DSI data were included into QTL mapping as a set of additional phenotypic traits.

### Genotyping

#### Mapping functional CGs on a consensus barley map

A consensus barley linkage map of SNP markers from BOPA1 (barley oligonucleotide pool assay 1; Close et al., 2009) and simple sequence repeat (SSR) markers, published earlier (Mikołajczak et al., 2016), was used in this study for the map enrichment with functional candidate genes (CG) and the construction of a new high-density, function barley map for the QTL analysis. Briefly, the consensus map was constructed for the MCam population and two other bi-parental RILs: LCam (derived from “Lubuski” × CamB) and GH (derived from “Georgie” × “Harmal”), produced from the crosses between European (drought susceptible) and Syrian (drought tolerant) spring barley cultivars. The map consisted of 819 markers, spanned 953.8 cM (an average resolution of 1.2 cM) and comprised of 13 linkage groups attributed to barley chromosomes 1H-7H. The map was uniformly covered with the markers, and was in good agreement with other integrated barley maps (Varshney et al., 2007; Close et al., 2009). The number of linkage groups exceeding the number of barley chromosomes was probably the effect of a diverse genetic background of parental genotypes, but it did not affect the marker order within the linkage groups, nor the CGs mapping and the overall quality of the map.

For the enrichment of the map, a group of 41 CGs potentially involved in drought response was selected based on the transcriptome analysis of “Maresi” and CamB genotypes under drought stress (Janiak et al., 2018). In addition, 23 genes encoding barley orthologs of drought tolerance-related genes, described in model species, were chosen (Supplementary Material S1). Publicly available genomic sequence information in GenBank (http://www.ncbi.nlm.nih.gov/genbank/) was utilized to identify barley genomic sequences of these CGs, so called functional CGs (Pflieger et al., 2001). The information on the structure and the chromosomal localizations of the CGs was derived from Ensembl Plants (http://plants.ensembl.org/index.html; ver. 082214v1).

For polymorphisms identification in the functional CG sequences, total genomic DNAs of two RIL populations, MCam and GH, and their parental forms, were extracted using a method by Doyle and Doyle (1987) with minor modifications. Primer pairs (Supplementary Material S2) for the PCR amplifications of CG fragments (800–1,200 bp) from genomic DNA of parental genotypes were designed with the software Primer 3 (http://bioinfo.ut.ee/primer3-0.4.0/). PCR products were sequenced from both ends at Genomed company (Warsaw, Poland; www.genomed.pl). The sequences assembly was done with the CodonCode Aligner software (CodonCode Corporation, Centerville, MA, USA).

Sequence polymorphisms (SNPs or insertions/deletions) identified in the CG sequences between the parental genotypes of RILs (“Maresi” vs. CamB or “Georgie” vs. “Harmal”) were subsequently genotyped within the adequate RIL population using one of the following methods: CAPS (cleaved amplified polymorphisms) or dCAPS (derived CAPS) for SNPs, and PCR for indels. In case of CAPS, the same primer pairs were used for the PCR amplification, then PCR products were cleaved with the appropriate restriction enzymes and visualized by 2.0% agarose gel electrophoresis. In the dCAPS, mismatch primers were designed with the dCAPS Finder 2.0. software (http://helix.wustl.edu/dcaps/dcaps.html) for nested-PCR amplification step, which followed the standard PCR amplification, then PCR products were cleaved with the appropriate restriction enzymes and visualized by 4.0% agarose gel electrophoresis. The indels were genotyped using the PCR amplifications with a newly designed primer pairs, followed by 4.0% agarose gel electrophoresis of the PCR products. Primer pairs designed for the methods of genotyping polymorphisms are given in Supplementary Material S2.

The functional CG linkage analysis was performed on the individual maps of the adequate mapping populations (MCam or GH), and was followed by construction of a new consensus, function map with the use all three maps and the software JoinMap 3.0 (Van Ooijen and Voorrips, 2001), according the procedure described previously (Mikołajczak et al., 2016). Marker order within each linkage group was check for the accordance with the other consensus barley maps (Varshney et al., 2007; Close et al., 2009). Final chromosome maps were drawn with the MapChart software (Voorrips, 2002).

#### QTL analysis

QTL analysis was performed with the MapQTL 5.0 software (Van Ooijen, 2004) for 40 physiological/biochemical traits measured under both water regimes (optimal water supply and drought) and for their DSI. QTLs were first mapped by interval mapping (IM), followed by multiple-QTL model (MQM), using the marker closest to the peak at each putative QTL as a cofactor. After performing a genome-wide permutation test with 1,000 iterations (Churchill and Doerge, 1994), a LOD (logarithm of the odds) thresholds from 2.8 to 3.5 (depending on the trait) were used to establish the presence of significant QTLs (*p* < 0.05). Confidence intervals for the QTLs were estimated based on two-LOD support interval, by taking two positions around the peak of the LOD profile, which had LOD-values by 2.0 lower than the maximum. The percentage of phenotypic variation (*R*^2^) explained by each QTL was calculated and a QTL was considered as major when it explained >10% of the phenotypic variation. The additive genetic effects were also calculated for the QTLs, and positive value indicated that the “Maresi” allele increased the trait value, whereas negative value indicated the decrease in the trait value caused by the “Maresi” allele. The QTLs which showed overlapping confidence intervals were clustered into hotspots.

### Identification of positional candidate genes within QTL confidence intervals

In order to identify the positional candidate genes (CG; i.e., closely linked genes localized within QTL regions; Pflieger et al., 2001), genetic and physical map integration was performed based on the positions of markers defining the boundaries of QTL confidence intervals (or the closest to them) in the genome. Nucleotide sequences of markers were mapped to the barley reference genomic sequence deposited in Ensembl Plants database (version 082214v1; http://plants.ensembl.org/index.html) using BLAST tool in order to project QTLs on the physical map (maximum *E*-value = 1E-100, minimum 95% identity of the sequence). Gene models found within the physical QTL intervals were retrieved using BioMart tool and grouped into the same four gene categories as the physiological/biochemical traits and corresponding QTL regions. This classification was based on the assumption that positional CGs identified within QTL regions related to a given trait category should be involved in the biological processes relevant to this trait category under the analyzed water regime.

### GO enrichment analysis

With the aim to identify GO terms (Biological Processes; BPs) associated with positional CGs and to determine the over-representation of a given GO term in an analyzed set of genes compared to the genome-wide background frequency, the GO enrichment analysis was performed using the PLAZA Monocots database version 3.0 (http://bioinformatics.psb.ugent.be/plaza/versions/plaza_v3_monocots; Proost et al., 2015). The significance of over-representation was determined using the hyper geometric distribution followed by the Bonferroni correction for multiple testing (corrected *p* ≤ 0.05). Furthermore, PLAZA was used to barley/Arabidopsis cross-species analysis in order to identify putative barley orthologs in the *Arabidopsis thaliana* genome. It was motivated by the extensive annotation features available for this model species.

## Results

### Phenotypic variation and correlations among traits

Descriptive statistics for physiological/biochemical traits evaluated in the MCam RIL population and parental genotypes is shown in Supplementary Material S3. The mean values of most of the measured parameters under both conditions: optimal water supply (C) and drought stress (D) as well as their stress indices (DSI), significantly differentiated both parental genotypes and varied among the RILs. Almost all traits were distributed normally within RIL population, and lines which exceeded the parental range of variation for different traits were observed. Significant transgressive segregation suggests a broad genetic diversity of parental genotypes and the polygenic inheritance of the investigated traits. Drought stress conditions caused a significant decrease of mean values for the majority of physiological parameters describing plant water status, specific energy fluxes, quantum efficiency ratios, photochemical activity of PSII and efficiency of gas exchange. On the other hand, an increase in proline and ethylene content, as well as in the activity of antioxidant enzymes and fat-soluble antioxidants, was observed. Analyzed genotypes varied in terms of carbohydrates content, both under control and drought conditions. Drought stress increased the accumulation of glucose, fructose and sucrose, whereas maltose and raffinose contents were diminished.

**Figure 1:**
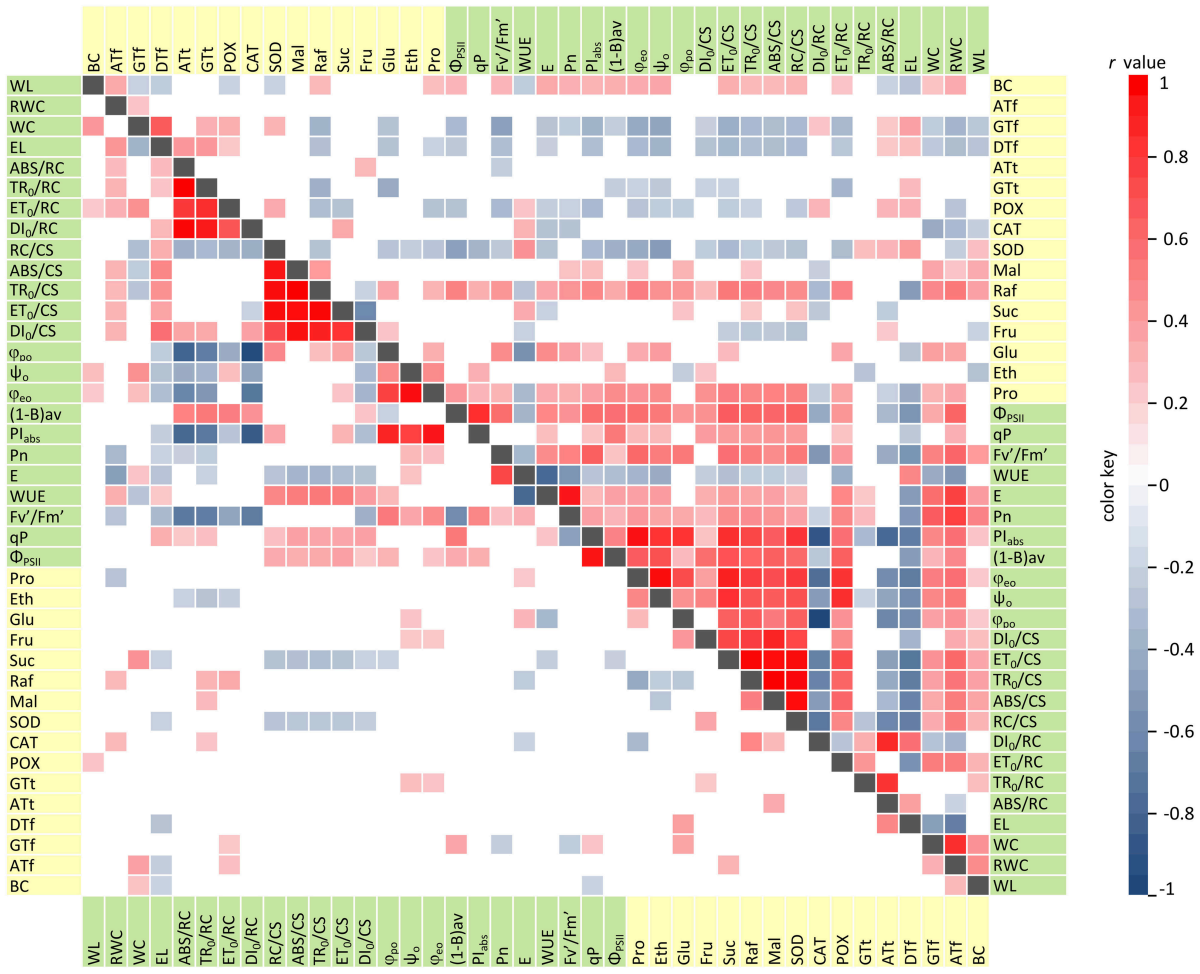
Correlation diagram for physiological (green) and biochemical (yellow) traits evaluated among RILs. Correlations under control conditions and drought stress are presented, respectively, in the lower and upper triangular. Statistically significant Pearson's coefficients (*r, p* < 0.05) are represented by colored matrix cells.

Under control conditions, strong significant correlations (|*r*| ≥ 0.5, *p* < 0.05) were exclusively observed among photosynthesis-related traits and parameters describing plant water status (Figure 1). The strongest positive correlations were revealed between: net photosynthesis (Pn) and transpiration (E); the photochemical quantum yield of PSII (Φ_PSII_) and the photochemical quenching (qP); the performance index per absorption (PI_abs_) and quantum efficiency ratios (ϕ_po_, ψ_o_, ϕ_eo_). Furthermore, three groups of parameters were found with significant internal correlations: specific energy fluxes (ABS, TR_0_, ET_0_, DI_0_) per excited leaf cross-section (CS), specific energy fluxes per reaction centre (RC), and quantum efficiency ratios (ϕ_po_, ψ_o_, ϕ_eo_). The strongest negative correlations were observed between: transpiration (E) and water use efficiency (WUE); and between ABS/RC, TR_0_RC, DI_0_/RC and the efficiency of excitation energy capture by open PSII reaction centres (F_v_'/F_m_'), PI_abs_, ϕ_po_, and ϕ_eo_.

Interestingly, the increasing number and magnitude of significant correlations, both among physiological parameters and between physiological and biochemical traits, was revealed under drought stress conditions (Figure 1). Photosynthetic efficiency parameters and plant water status traits were positively correlated in the majority of cases. The strongest relationships, not identified under control conditions, were observed between: the RWC and Φ_PSII_, Pn, E, ${\text{F}}_{\text{v}}^{\prime }/{\text{F}}_{\text{m}}^{\prime }$; as well as Φ_PSII_ and the number of active RCs per CS (RC/CS), specific energy fluxes (ABS, TR_0_, ET_0_,) per CS, PI_abs_, and (1-B)av. Only three parameters, i.e., electrolyte leakage (EL), ABS/RC, and DI_0_/RC were strongly and negatively correlated with all of the other physiological traits. This indicates drought-induced increase of the permeability of cell membranes, inactivation of a part of RC pool and increase of the antenna size. Under drought stress, in contrast to optimal water supply, we observed numerous and mainly negative correlations between the physiological and biochemical parameters (in particular, for SOD, POX, γ- and α-tocopherols activities). Taken together, these results revealed the changes in cellular metabolism during water deficit which activated the processes enhancing barley adaptation capacity to unfavorable conditions and prevented from a negative impact of drought stress on photosynthesis efficiency.

### Quantitative trait loci identification

QTL analysis was performed using our previously published high-density SNP and SSR-based consensus genetic map (Mikołajczak et al., 2016) which was enriched with 64 functional CGs including differentially expressed genes (DEGs) derived from the transcriptome analysis of parental genotypes under drought stress and barley orthologs of drought tolerance-related genes described in model species (Supplementary Material S1). The newly constructed barley function map consists of 875 loci and spans 941.86 cM with an average resolution of 1.1 cM. Based on comparison with physical map we estimated that it covers about 95% of the barley genome. This provides an excellent framework for QTL identification. In order to extensively evaluate the response of the analyzed genotypes to drought, both, the direct values of the 40 physiological and biochemical parameters measured under control conditions and drought stress, as well as the relative values (DSI) for each trait, were used in QTL mapping.

**Figure 2:**
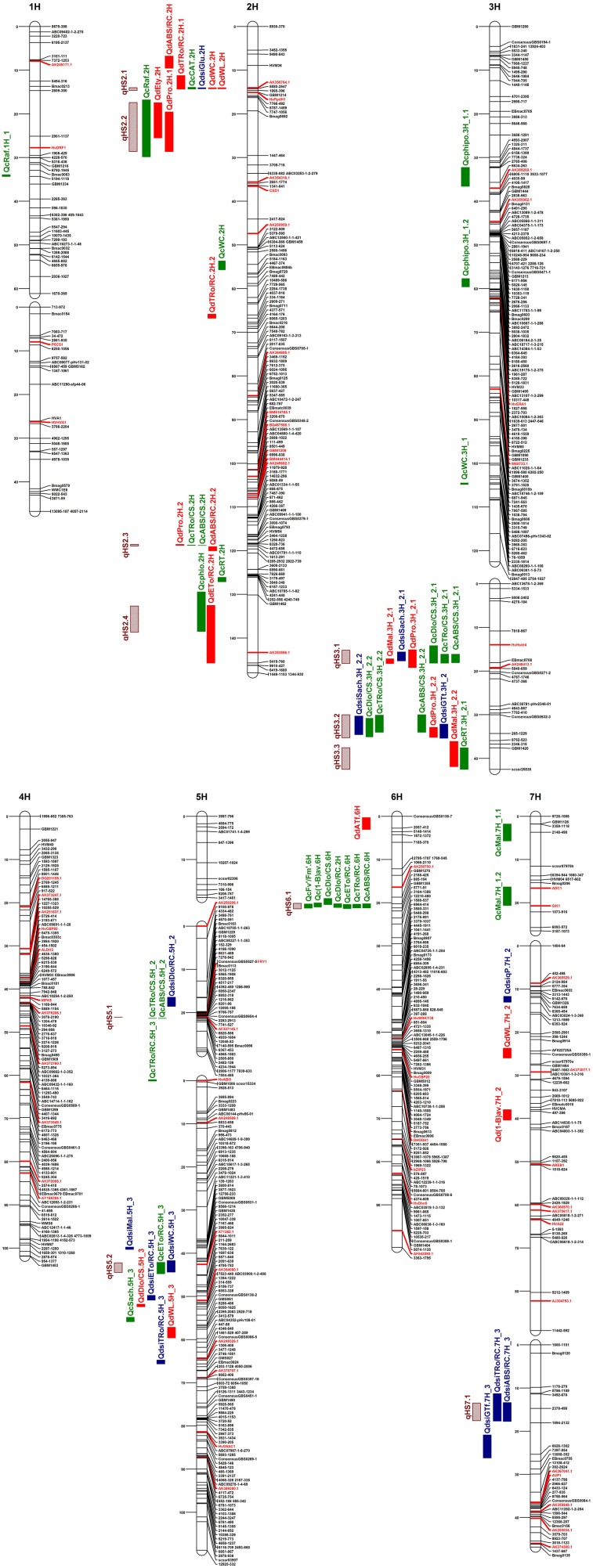
High-density consensus function map of barley with the positions of QTLs for physiological and biochemical traits related to the drought stress response. Markers are given on the right side of the linkage groups. Functional candidate genes are given in red. Bars represent intervals associated with QTLs for: drought stress (red), control conditions (green), stress indices (blue), and QTL hotspots (patterned).

A total of 64 QTLs for 25 drought tolerance-related traits that describe plant water status, photosynthetic efficiency, osmoprotectant, and hormone content, as well as activity and accumulation of antioxidants were identified among all of the chromosomes, except 4H (Figure 2). The number of detected QTLs varied from 1 to 4, depending on the trait and the experiment variant (C/D/DSI). The highest number of QTLs were positioned on chromosomes 2H and 3H (18 and 17, respectively), and 5H (12). Eight regions for QTLs were mapped on chromosomes 6H and 7H, whereas chromosome 1H contained a single QTL. The maximum LOD scores estimated in the QTL confidence intervals ranged from 3.0 to 20.76, while the phenotypic variation explained by an individual QTL varied from 6.7 to 87.5%. Chromosome 3H contained regions that accounted for most of the observed phenotypic variation in the investigated parameters (14 QTLs with at least 30% of *R*^2^). Favorable alleles which had positive effects on the variation in analyzed traits came from both parental genotypes, “Maresi” and CamB.

Under control conditions, the QTL analysis revealed 32 chromosomal regions for 17 traits (Table 2). Most (22) QTLs were found to determine photosynthetic efficiency. The others were related to the plant water status traits (4), raffinose content (2), maltose content (2), sucrose content (1), and catalase activity (1). About a half of the identified QTLs under control conditions explained a large proportion of the observed phenotypic variation (*R*^2^ ≥ 30%). Chromosomal regions with the highest *R*^2^ values (exceeding 60%) were mapped for RWC, TR_0_/CS, DI_0_/CS and maltose content. Furthermore, two QTLs located on chromosome 7H, which explained more than 85% of the observed phenotypic variation in maltose content, were characterized by the highest LOD scores (>20.0) among all the QTLs revealed in the analyzed RIL population. Three functional CGs, co-segregating with the maximum LOD scores within confidence intervals of the QTLs mapped under control conditions, were found. X71362.1 gene, encoding a member of the dehydrin family, was associated with the QTL for ET_0_/RC on chromosome 5H. On the same chromosome, *HvABI5* gene, involved in plant response to abscisic acid, was located in the QTL confidence interval for TR_0_/RC. Finally, the QTL for maltose content mapped on chromosome 7H co-segregated with barley ortholog of *ADC1* gene which encodes arginine decarboxylase.

**Table 2:** Summary of QTLs detected for 17 physiological and biochemical traits in the MCam population under control conditions.

**Trait**	**Control conditions**						
	**QTL name**	**Chr**.	**Nearest marker**	**LOD**	**Confidence interval [cM]**	**Additive effect**	***R^2^* [%]**
**PLANT WATER STATUS**							
RWC	QcRWC.2H	2H	252-556	7.44	126.13–127.13	−0.463	**84.4**
	QcRWC.3H_2.1	3H	scssr25538	7.34	37.59–42.52	9.432	**83.8**
WC	QcWC.2H	2H	5184-1163	3.34	54.46–56.46	−0.338	13.1
	QcWC.3H_1	3H	5260-462	3.11	104.52–104.83	0.358	12.1
**PHOTOSYNTHETIC EFFICIENCY**							
ABS/RC	QcABS/RC.6H	6H	885-104	7.01	20.25–21.22	−0.182	**34.8**
TR_0_/RC	QcTRo/RC.5H_3	5H	HvABI5	3.46	0–0.32	0.051	10.5
	QcTRo/RC.6H	6H	2188-425	8.53	20.25–21.22	−0.108	**30.7**
ET_0_/RC	QcETo/RC.5H_3	5H	X71362.1	3.75	41.96–44.59	−0.024	11.9
	QcETo/RC.6H	6H	885-104	7.55	20.25–21.22	−0.048	**30.0**
DI_0_/RC	QcDlo/RC.2H	6H	885-104	5.62	20.25–21.22	−0.072	**30.0**
ABS/CS	QcABS/CS.2H	2H	2464-1228	3.35	118.27–118.27	787.930	10.0
	QcABS/CS.3H_2.1	3H	EBmac0708	6.51	16.02–18.08	1907.390	**55.6**
	QcABS/CS.3H_2.2	3H	ConsensusGBS0632-3	**11.09**	30.01–34.01	−1722.250	**59.1**
	QcABS/CS.5H_2	5H	Bmac0096	4.05	21.56–21.56	−192.893	11.6
TR_0_/CS	QcTRo/CS.2H	2H	2464-1228	4.28	118.27–118.32	637.155	10.7
	QcTRo/CS.3H_2.1	3H	EBmac0708	7.59	16.02–18.08	1558.190	**57.8**
	QcTRo/CS.3H_2.2	3H	ConsensusGBS0632-3	**12.39**	30.01–34.01	−1448.690	**60.5**
	QcTRo/CS.5H_2	5H	Bmac0096	4.25	21.56–21.56	−152.128	6.7
DI_0_/CS	QcDlo/CS.3H_2.1	3H	EBmac0708	9.04	14.02–18.08	331.108	**63.8**
	QcDlo/CS.3H_2.2	3H	265-1229	7.99	31.01–35.29	−359.451	**64.3**
	QcDlo/CS.6H	6H	2188-425	3.03	18.74–20.25	−55.813	6.7
ϕ_po_	Qcphi_po.3H_1.1	3H	6634-263	4.43	32.33–36.69	−0.006	17.0
	Qcphi_po.3H_1.2	3H	ABC19175-1-2-375	5.66	57.79–59.70	0.008	23.5
ψ_o_	Qcpsi_o.2H	2H	GBM1462	3.15	129.13–138.17	0.009	17.2
(1-B)av	Qc(1-B)av.6H	6H	885-104	5.8	20.25–21.22	−0.470	**30.0**
F_v_'/F_m_'	QcFv'/Fm'.6H	6H	885-104	3.85	20.25–21.22	0.018	23.3
**OSMOPROTECTANT AND HORMONE CONTENT**							
Suc	QcSuc.5H_3	5H	GMS061	4.68	55.55–56.54	−6.558	21.1
Raf	QcRaf.1H_1	1H	5194-1118	3.84	33.95–34.34	−0.339	16.2
	QcRaf.2H	2H	Bmag0692	9.01	16.28–29.33	−0.458	**34.8**
Mal	QcMal.7H_1.1	7H	2148-498	**20.76**	1.79–5.69	−14.320	**87.5**
	QcMal.7H_1.2	7H	ADC1	**20.75**	16.36–20.66	14.387	**85.4**
**ACTIVITY AND ACCUMULATION OF ANTIOXIDANTS**							
Cat	QcCAT.2H	2H	1865-396	7.29	14.23–14.63	−0.003	**30.3**

*Functional candidate genes that co-segregated with the maximum LOD scores within confidence intervals of mapped QTLs are underlined; LOD ≥ 10 and R^2^ ≥ 30 are in bold. R^2^, percentage of phenotypic variation explained by individual QTL*.

The QTL analysis performed for the same physiological/biochemical parameters of RILs grown under drought stress conditions showed the presence of 19 QTL regions for 11 traits (Table 3). All detected QTLs were declared as the major ones, as they explained more than 10% of the phenotypic variation of the particular traits. The highest number of QTLs was identified for photosynthetic efficiency parameters (7), as well as osmoprotectant content (6). The remaining QTLs were found to determine plant water status (4), ethylene content (1), and α-tocopherol activity (1). The highest *R*^2^ values and LOD scores described the chromosomal regions involved in the proline and maltose accumulation. Drought-induced proline level was found to be controlled by three major QTLs mapped on chromosomes 2H and 3H, which explained between 73.6 and 77.5% of the observed phenotypic variation, but an additional locus with a smaller effect was identified on chromosome 2H. In addition, two QTLs located on chromosome 3H, involved in maltose content regulation, were characterized by LOD scores above 18.0 and *R*^2^ values that exceeded 85%. Two differently expressed functional CGs were found to be associated with the chromosomal regions for fast kinetics of chlorophyll *a* fluorescence parameters, located on chromosome 2H. Gene AK356764.1 that encodes transketolase co-localized with the QTL for TR_0_/RC, whereas gene AK353596.1 encoding a member of the peroxidase family showed coincidence with the LOD peak region for ET_0_/RC.

**Table 3:** Summary of QTLs detected for 11 physiological and biochemical traits in the MCam population under drought stress.

**Trait**	**Drought stress conditions**						
	**QTL name**	**Chr**.	**Nearest marker**	**LOD**	**Confidence interval [cM]**	**Additive effect**	***R^2^* [%]**
**PLANT WATER STATUS**							
WL	QdWL.2H	2H	5880-2547	6.84	14.03–14.23	−0.202	21.9
	QdWL.5H_3	5H	3412-579	4.15	57.16–9.59	0.136	12.9
	QdWL.7H_2	7H	ConsensusGBS0356-1	3.15	23.72–25.99	−0.121	10.6
WC	QdWC.2H	2H	5880-2547	3.82	14.03–14.23	−0.323	17.2
**PHOTOSYNTHETIC EFFICIENCY**							
ABS/RC	QdABS/RC.2H.1	2H	9490-843	3.97	5.42–8.31	−0.085	17.3
	QdABS/RC.2H.2	2H	285-2932	4.09	119.60–120.57	−0.166	17.7
TR_0_/RC	QdTRo/RC.2H.1	2H	AK356764.1	3.55	10.92–14.03	−0.051	13.4
	QdTRo/RC.2H.2	2H	2809-271	4.69	66.78–67.71	−0.047	16.9
ET_0_/RC	QdETo/RC.2H	2H	AK353596.1	3.85	132.17–145.34	0.041	20.5
DI_0_/CS	QdDlo/CS.5H_3	5H	5156-737	3.89	51.79–52.38	44.152	17.5
(1-B)av	Qd(1-B)av.7H_2	7H	497-386	3.33	37.97–40.35	−0.354	18.7
**OSMOPROTECTANT AND HORMONE CONTENT**							
Pro	QdPro.2H.1	2H	Bmag0692	6.36	19.28–28.33	3.114	12.2
	QdPro.2H.2	2H	6328-736	**10.62**	118.44–118.63	−7.080	**75.2**
	QdPro.3H_2.1	3H	EBmac0708	**10.53**	15.02–19.08	−11.964	**73.6**
	QdPro.3H_2.2	3H	265-1229	**11.58**	33.01–35.29	20.832	**77.5**
Mal	QdMal.3H_2.1	3H	EBmac0708	**18.38**	17.02–18.08	1.370	**85.8**
	QdMal.3H_2.2	3H	GBM1420	**18.44**	36.75–42.52	−1.533	**85.9**
Eth	QdEth.2H	2H	Bmag0692	3.66	18.28–26.33	0.084	18.5
**ACTIVITY AND ACCUMULATION OF ANTIOXIDANTS**							
ATf	QdATf.6H	6H	ConsensusGBS0136-7	4.36	0–2.75	−0.019	20.0

*Functional candidate genes that co-segregated with the maximum LOD scores within confidence intervals of mapped QTLs are underlined; LOD ≥ 10 and R^2^ ≥ 30 are in bold. R^2^, percentage of phenotypic variation explained by individual QTL*.

All 13 QTLs mapped for stress indices (DSI) of 11 parameters were classified as the major ones (Table 4). Most of them were detected for photosynthetic efficiency and osmoprotectant content (6 and 4, respectively), similarly to the drought stress conditions. The other QTLs were identified for leaf water content (1) and tocochromanols activity (2). The highest proportion of observed phenotypic variation, ranging from 60.8 to 82.1%, was explained by two QTLs positioned on chromosome 3H: for sucrose and for γ-tocotrienol stress indices. Two functional CGs from the transcriptome analysis of “Maresi” and CamB co-segregated with maximum LOD scores within the QTL confidence intervals, identified on chromosome 5H. Dehydrin gene (X71362.1) co-localized with the QTLs for leaf water content stress index, while AK356764.1 gene that encodes cysteine protease was associated with the QTL for TR_0_/RC stress index.

**Table 4:** Summary of QTLs for stress indices (DSI) for 11 physiological and biochemical traits in the MCam population.

**Trait**	**Stress indices (DSI)**						
	**QTL name**	**Chr**.	**Nearest marker**	**LOD**	**Confidence interval [cM]**	**Additive effect**	***R*^2^ [%]**
**PLANT WATER STATUS**							
WC	QdsiWC.5H_3	5H	X71362.1	4.98	41.96–44.59	6.097	23.6
**PHOTOSYNTHETIC EFFICIENCY**							
ABS/RC	QdsiABS/RC.7H_3	7H	2378-498	3.68	13.39–17.73	3.663	16.7
TR_0_/RC	QdsiTRo/RC.5H_3	5H	3477-1248	3.84	64.75–65.63	−2.242	14.4
	QdsiTRo/RC.7H_3	7H	2378-498	4.19	11.39–17.73	2.399	16.5
ET_0_/RC	QdsiETo/RC.5H_3	5H	AK364080.1	3.57	49.85–50.94	3.310	16.2
DI_0_/RC	QdsiDlo/RC.5H_2	5H	4570-591	3.61	16.46–18.57	15.007	18.1
qP	QdsiqP.7H_2	7H	ABC03024-1-3-368	3.00	11.98–13.36	4.848	15.5
**OSMOPROTECTANT AND HORMONE CONTENT**							
Glu	QdsiGlu.2H	2H	5880-2547	5.81	14.03–14.23	−107.193	25.7
Suc	QdsiSuc.3H_2.1	3H	EBmac0708	4.88	16.02–18.08	405.162	**60.8**
	QdsiSuc.3H_2.2	3H	265-1229	4.29	31.01–35.29	−400.512	**54.6**
Mal	QdsiMal.5H_3	5H	GBM5008	3.28	37.50–38.03	17.274	15.5
**ACTIVITY AND ACCUMULATION OF ANTIOXIDANTS**							
GTf	QdsiGTf.7H_3	7H	6628-1302	3.09	21.05–26.34	60.182	16.0
GTt	QdsiGTt.3H_2	3H	265-1229	**14.1**	32.01–35.29	−248.340	**82.1**

*Functional candidate genes that co-segregated with the maximum LOD scores within confidence intervals of mapped QTLs are underlined; LOD ≥ 10 and R^2^ ≥ 30 are in bold. R^2^, percentage of phenotypic variation explained by individual QTL*.

### Co-location of QTLs for different traits

The positioned QTLs were not distributed evenly in the barley genome, and they clearly tend to be clustered in the particular chromosome regions. Based on their overlapping confidence intervals, 11 hotspots were identified that contained together more than 60% of mapped QTLs for different traits (Table 5, Figure 2). The highest number of hotspots (4), that combined the QTLs from all investigated trait categories analyzed in all experiment variants (C/D/DSI), was located on chromosome 2H, followed by three hotspots which contained 2–6 overlapping QTL confidence intervals detected on chromosome 3H. Two hotspots were localized on chromosome 5H, each of them for two physiological parameters. Then, a single genomic region on chromosome 6H enclosed 7 QTLs for photosynthetic efficiency under control water conditions. The last hotspot, that included 2 overlapping QTLs for DSI of fast kinetics of chlorophyll *a* fluorescence parameters, was identified on chromosome 7H.

**Table 5:** Main characteristics of QTL hotspot regions.

**Hotspot**	**Trait**	**Experiment variant**	**QTL name**	**Chromosome**	**Confidence interval [cM]**
qHS2.1	TR_0_/RC	D	QdTRo/RC.2H.1	2H	10.92–14.63
	WC	D	QdWC.2H		
	WL	D	QdWL.2H		
	CAT	C	QcCAT.2H		
	Glucose	DSI	QdsiGlu.2H		
qHS2.2	Proline	D	QdPro.2H.1	2H	16.28–29.33
	Ethylene	D	QdEth.2H		
	Raffinose	C	QcRaf.2H		
qHS2.3	Proline	D	QdPro.2H.2	2H	118.27–118.63
	TR_0_/CS	C	QcTRo/CS.2H		
	ABS/CS	C	QcABS/CS.2H		
qHS2.4	ET_0_/RC	D	QdETo/RC.2H	2H	129.13–145.34
	ψ_o_	C	Qcpsi_o.2H		
qHS3.1	Maltose	D	QdMal.3H_2.1	3H	14.02–19.08
	Proline	D	QdPro.3H_2.1		
	DI_0_/CS	C	QcDlo/CS.3H_2.1		
	TR_0_/CS	C	QcTRo/CS.3H_2.1		
	ABS/CS	C	QcABS/CS.3H_2.1		
	Sucrose	DSI	QdsiSuc.3H_2.1		
qHS3.2	Proline	D	QdPro.3H_2.2	3H	30.01–35.29
	DI_0_/CS	C	QcDlo/CS.3H_2.2		
	TR_0_/CS	C	QcTRo/CS.3H_2.2		
	ABS/CS	C	QcABS/CS.3H_2.2		
	γ-tocotrienol	DSI	QdsiGTte.3H_2		
	Sucrose	DSI	QdsiSuc.3H_2.2		
qHS3.3	Maltose	D	QdMal.3H_2.2	3H	36.75–42.52
	RWC	C	QcRWC.3H_2.1		
qHS5.1	TR_0_/CS	C	QcTRo/CS.5H_2	5H	21.56
	ABS/CS	C	QcABS/CS.5H_2		
qHS5.2	ET_0_/RC	C	QcETo/RC.5H_3	5H	41.96–44.59
	WC	DSI	QdsiWC.5H_3		
qHS6.1	F_v_'/F_m_'	C	QcFv'/Fm'.6H	6H	18.74–21.22
	(1-B)av	C	Qc(1-B)av.6H		
	DI_0_/CS	C	QcDlo/CS.6H		
	DI_0_/RC	C	QcDlo/RC.2H		
	ET_0_/RC	C	QcETo/RC.6H		
	TR_0_/RC	C	QcTRo/RC.6H		
	ABS/RC	C	QcABS/RC.6H		
qHS7.1	TR_0_/RC	DSI	QdsiTRo/RC.7H_3	7H	11.39–17.73
	ABS/RC	DSI	QdsiABS/RC.7H_3		

*D, drought stress; C, control conditions; DSI, stress index*.

### QTL projection on barley genome

**Figure 3:**
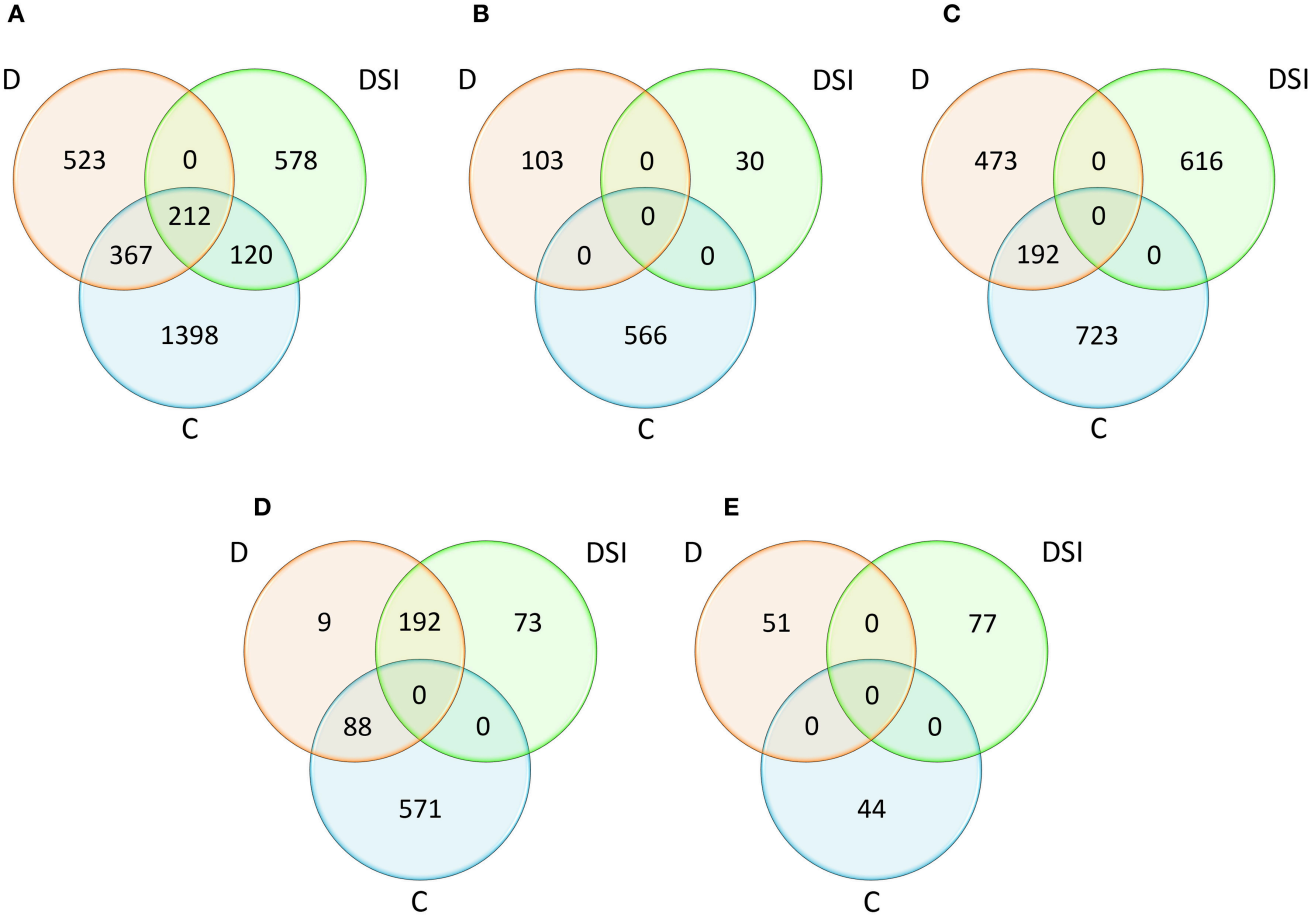
Comparative analysis of the numbers of positional candidate genes identified within QTL confidence intervals with respect to the experiment variants (C, D, DSI) for all traits **(A)** and for the considered trait categories: plant water status **(B)**, photosynthetic efficiency **(C)**, osmoprotectant and hormone content **(D)**, activity and accumulation of antioxidants **(E)**. D, drought stress; C, control conditions; DSI, stress index.

One of the main goals of this study was to identify the positional candidate genes (CGs) within QTL confidence intervals, followed by the exploration of their putative functions related to drought tolerance. We used the availability of the barley reference genome sequence to search for positional CGs in the chromosomal regions corresponding to the confidence intervals of the QTLs determined on the consensus map. The integration of the genetic and physical map led to the identification of overall 3,198 positional candidate genes that could be responsible for the variation in the analyzed traits (Supplementary Material S4; Figure 3A). Among them, there were the CGs (1,101) underlying the drought response-specific QTLs, i.e., those QTLs which were detected under drought stress conditions and for the stress indices. The number of genes included in a particular QTL region for all analyzed traits varied between 1 and 550, and was correlated with the confidence interval size, whereas the total ratio between physical and genetic distances was 2.11 Mbp/cM.

Comparative analysis of the number of the positional CGs classified into four trait categories, as well as three analyzed experiment variants (C/D/DSI) was shown on Figures 3B–E. When considering plant water status and antioxidant activity, 699 and 172 genes, respectively, were specific to the analyzed experiment variants. Altogether, 2,004 positional CGs were identified for photosynthetic efficiency, and approximately 30% of CGs involved in this process under drought stress (192 out of 665) were also detected in the control conditions. Even larger overlap was observed among 933 genes corresponding to the osmoprotectant and hormone content. Approximately 30% of positional CGs identified under drought stress (88 out of 289) were also detected under control conditions, while as much as 66% (192) were common with stress indices of the analyzed traits. Only 9 genes were identified exclusively under drought stress within this trait category.

### QTL candidate genes prioritization

Due to the limited resolution of QTL mapping which resulted in a high number of identified positional candidate genes, we applied Gene Ontology (GO) enrichment approach to: (1) predict the functional ontologies associated with positional CGs, (2) to determine the over-representation of certain GO terms in the analyzed gene sets compared to the genome-wide background frequency, (3) to reduce the number of CGs to potentially casual genes responsible for the variation in the considered traits.

**Figure 4:**
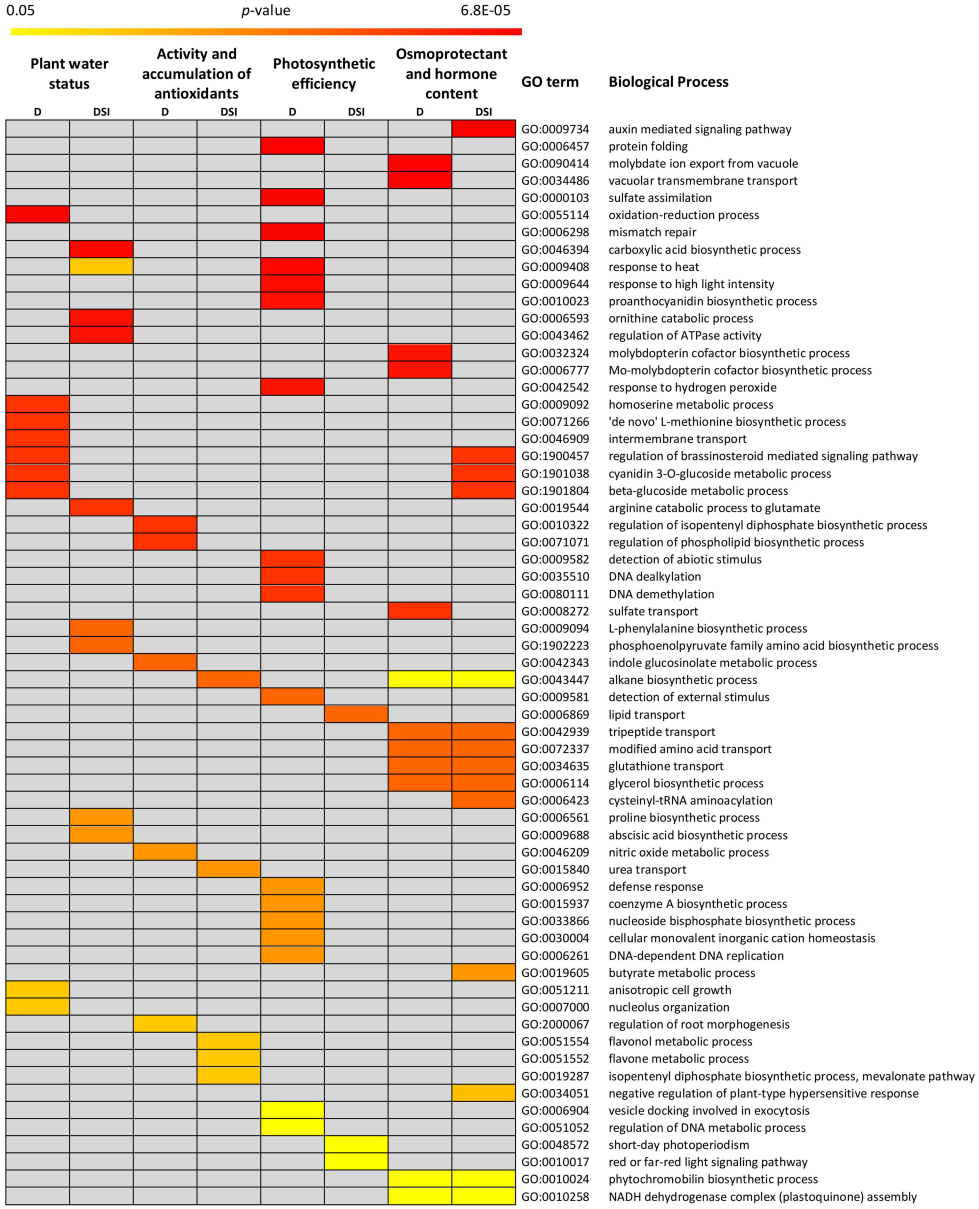
Biological processes significantly over-represented in the gene sets for the analyzed trait categories: under drought stress (D) and for stress indices (DSI).

As for the gene sets detected for the analyzed trait categories under optimal water supply, the functional annotation showed their participation in Biological Processes (BP) clearly relevant for the proper shaping of plant growth and development at the morphological, anatomical and physiological levels. They included e.g., post-embryonic organ development, carbohydrate mediated signaling, pentose metabolic process, protein phosphorylation, stomatal movement, defense response, as well as biosynthesis and transport of various compounds (Supplementary Material S5). In order to gain an insight into molecular mechanisms of drought stress response in barley, the special attention was paid to 1,101 positional CGs underlying drought response-specific QTLs. The GO enrichment analysis resulted in narrowing down this number to 143 prime candidates involved in the significantly over-represented BPs which were closely related to the exposition of the analyzed genotypes to drought stress (Figure 4). Some of them are described as unknown in existing annotations and assessment of their potential engagement in drought response in barley requires further exploration of their biological functions.

#### CGs for plant water status

The GO enrichment in the set of positional CGs identified for plant water status under drought stress, revealed 17% of them to be involved in the oxidation-reduction process (Supplementary Material S6). A detailed analysis showed that two barley genes, MLOC_5505 and MLOC_69302, encoding enzymes belonging to the dehydrogenase and peroxidase families, respectively, were identified in qHS2.1 hotspot which included the overlapping QTLs for leaf water content and water loss under drought, as well as glucose content stress index and catalase activity under control water conditions. The other genes of this GO category are: MLOC_6299 (an ortholog of Arabidopsis AT5G65110, involved in biosynthesis of long chain fatty acid), MLOC_4579 (flavonol synthase), and MLOC_71498 (an ortholog of Arabidopsis gene AT4G25650, encoding translocation channel at the inner envelope membrane of chloroplasts). All these genes were detected in the QTL confidence interval for water loss, on chromosome 7H. The remaining highly enriched group of genes were annotated as intermembrane transport (MLOC_52403 encoding vesicle-associated membrane protein), homoserine metabolic process and “*de novo*” L-methionine biosynthetic process (MLOC_71910, cystathionine gamma), cyanidin 3-O-glucoside metabolic process and beta-glucoside metabolic process (MLOC_36976, UDP-glucosyl transferase), and regulation of brassinosteroid mediated signaling pathway (MLOC_72613, L-type lectin-domain containing receptor kinase III). Among the CGs for water content stress index, the most enriched Biological Process was carboxylic acid biosynthesis. A detailed analysis of the genes classified into this BP category revealed two barley genes: MLOC_18300 (a member of NCED-related gene family) encoding the key enzyme involved in ABA biosynthesis, and MLOC_53947 for putative plastid pyruvate dehydrogenase. The others significantly over-represented GO terms were: proline biosynthesis process, ornithine catabolic process, arginine catabolic process to glutamate (MLOC_35821, ornithine aminotransferase), regulation of ATPase activity, and response to heat (MLOC_37449, protein DnaJ).

#### CGs for photosynthetic efficiency

The functional annotation analysis of the positional CGs identified for photosynthetic efficiency under drought stress showed that the majority of genes belong to the GO categories of protein folding, response to heat, response to high light intensity, and response to hydrogen peroxide (Supplementary Material S7). They include genes located mostly on chromosomes 2H and 7H that encoded heat shock proteins (MLOC_568, MLOC_31567, MLOC_41281) and chaperonins (MLOC_51927 and MLOC_54083). Some of them are involved in folding of RuBisCO, the major enzyme at the first step of carbon fixation in the Calvin cycle. A particularly interesting GO category comprised three genes mapped on chromosomes 2H and 5H (MLOC_15256, MLOC_19075, MLOC_38928), encoding ATP sulfurylases involved in the sulfate assimilation pathway. The other highly enriched Biological Process among CGs for photosynthetic efficiency in response to drought was detection of abiotic stimulus. Two genes, one encoding phytochrome A (MLOC_824) and the other involved in the chromatin remodeling in the response to environmental cues (MLOC_60235) were found within this category. The most enriched GO terms of the CGs for photosynthetic efficiency DSI included three groups. The first group consisted of the over-represented BPs related to DNA dealkylation and DNA demethylation (MLOC_53029 and MLOC_11707, encoding DNA repair proteins). Another highly represented GO category was lipid transport. Here, MLOC_51456 encoding a non-specific lipid-transfer protein was found. The BPs belonging to the last group were related to short-day photoperiodism that included MLOC_6879 (an ortholog of Arabidopsis gene AT3G24440, *VERNALIZATION INSENSITIVE 3-LIKE 1*) and MLOC_19228 (an ortholog of Arabidopsis gene AT5G46210, *CULLIN4*). Red or far-red light signaling pathway was another overrepresented BP from this group, including MLOC_20045, encoding nucleoside diphosphate kinase and MLOC_53845, an ortholog of Arabidopsis gene *PFT1*, encoding phytochrome and flowering time regulatory protein).

#### CGs for osmoprotectant and hormone content

The majority of drought-specific CGs for the osmoprotectant and hormone content category were common for drought stress conditions and stress indices (DSI). According to the GO enrichment, they are involved in tripeptide and modified amino acid transport, glutathione transport (MLOC_174, a member of Crt-like transporters), glycerol biosynthetic process (MLOC_40292, glycerol-3-phosphatase 1), and phytochromobilin metabolic process (MLOC_70465, phytochromobilin oxidoreductase; Supplementary Material S8). These genes encode proteins located in chloroplasts and they were mapped in qHS3.1 hotspot which included the overlapping QTLs for sucrose content stress index, as well as for proline and maltose content under drought stress. Among CGs for this category, GO enrichment analysis led to the identification of two barley genes exclusive for drought stress, which were positioned in qHS3.2 hotspot. MLOC_65646 is a putative ortholog of Arabidopsis gene AT5G20990, encoding molybdopterin biosynthesis protein, which is involved in molybdenum cofactor biosynthesis, whereas MLOC_22343, encoding molybdate transporter showed enrichment in BP categories related to vacuolar transmembrane transport, molybdate ion export from vacuole, and sulfate transport. The most represented category of BPs among the CGs for osmoprotectant and hormone content stress indices was auxin mediated signaling pathway. One of the genes involved in this process was MLOC_58506, encoding a member of the small auxin up-regulated RNA family. It was mapped on chromosome 5H in QTL confidence interval for maltose content stress index. Other highly enriched GO categories were the same as for the plant water status under drought stress: cyanidin 3-O-glucoside metabolic process, beta-glucoside metabolic process (MLOC_36976, UDP-glucosyl transferase), and regulation of brassinosteroid mediated pathway (MLOC_72613, L-type lectin-domain containing receptor kinase III).

#### CGs for activity and accumulation of antioxidants

For the activity and accumulation of antioxidants trait category, a single QTL for α-tocopherol content under water-deficiency conditions was identified on chromosome 6H. The GO enrichment analysis among CGs for α-tocopherol led to the identification of over-represented BP categories, such as: regulation of isopentenyl diphosphate biosynthetic process, regulation of phospholipid biosynthetic process, nitric oxide metabolic process, and indole glucosinolate metabolic process (Supplementary Material S9). In-depth analysis showed MLOC_63263, encoding a chloroplastic/mitochondrial NO-associated protein, and MLOC_57100, encoding O-methyltransferase which is involved in flavonol biosynthesis. The analysis of the GO annotations of the positional CGs for antioxidant activity DSI revealed their involvement in: flavonol metabolic process, flavone metabolic process (MLOC_73233, O-methyltransferase), and urea transport (MLOC_58872, an ortholog of Arabidopsis gene AT4G01470, encoding tonoplast intrinsic protein functions as water and urea channel). Furthermore, the both selected genes for antioxidant activity stress indices were identified in qHS3.2 hotspot which overlapped 6 QTLs, among them for γ-tocotrienol and sucrose stress indices, as well as proline content under drought stress.

## Discussion

The identification of genes underlying particular QTLs, followed by the elucidation of their molecular functions, is one of the paramount challenges in the modern plant genetics, as more than 2500 studies on QTL analysis in crop plants have been published so far (Kumar et al., 2017). Most studies have focused on exploiting the highly saturated molecular function maps to co-localize QTL regions with the genetic markers, based on within-gene polymorphisms (Diab et al., 2008; Sehgal et al., 2012; Li W. T. et al., 2013; de Miguel et al., 2014; Mikołajczak et al., 2016). In a standard strategy, after fine mapping of a QTL, a positional cloning of the QTL has been proposed to identify a gene or genes underlying a complex trait (Kumar et al., 2017). Although, there are examples of successful positional cloning of QTLs (Salvi and Tuberosa, 2007; Collins et al., 2008; Mir et al., 2012), this procedure remains difficult and cumbersome, especially for large genomes. This could explain a small number of cloned QTLs in barley with only a few examples related to abiotic stress tolerance, such as boron (Sutton et al., 2007), and aluminum toxicity (Furukawa et al., 2007) or freezing tolerances (Francia et al., 2007).

Recently, the availability of complete genome sequences for a growing number of plant species gave the opportunity to gain an insight into the physical gene space, resulting in the straightforward searching for all possible positional candidate genes associated with QTL confidence intervals (Monclus et al., 2012; Bargsten et al., 2014; Correa et al., 2014). Therefore, an inherent bottleneck is an effective selection of the most promising CGs within QTL regions, typically including tens to hundreds of genes, most of them unrelated to the trait of interest. Various methods to prioritize CGs within the QTL regions for the target trait can be combined, including the approach based on CGs overrepresentation in the biological processes, the analysis of their differential expression at transcriptome and/or proteome levels or functional annotation of CGs based on their orthologs in related or model species (Monclus et al., 2012; Bargsten et al., 2014; Kumar et al., 2017).

Our study presents the comprehensive multistep approach for elucidation of the genetic basis of drought response mechanisms in barley. Firstly, the identification of precise chromosomal regions underlying physiological and biochemical indicators of the drought tolerance was accomplished by merging the extensive analysis of these parameters under drought stress and classical quantitative genetic approach, performed with the use of strict threshold criteria and a high-density function map. Next, the anchoring of drought response-specific QTLs to the reference barley genome sequence was performed, after the thorough evaluation of QTL data. Finally, a huge number of identified positional CGs was reduced to potentially causative genes underlying the analyzed traits, using a Gene Ontology enrichment approach, followed by additional prioritization tools. Below, the most important results of the particular steps of the study are discussed.

### Reliability and accuracy of QTL mapping

QTL analysis in this study identified a total of 64 QTLs for 25 out of 40 physiological and biochemical traits measured under the optimal and drought stress conditions (absolute values), as well as for their DSI (relative values). The identified QTLs showed LOD scores ranging from 3.0 to 20.8, and explained 6.7 to 87.5% of observed variation (*R*^2^). The applied LOD threshold of 3.0 to declare a presence of the QTL ensured the detection of reliable and significant QTLs involved in the formation of analyzed traits and the exclusion of small effect, tentative QTLs (Salvi and Tuberosa, 2015; Kumar et al., 2017). Using the same criterion (LOD > 3.0), Liu et al. (2017) identified 11 significant QTLs for 6 traits, out of a total of 15 stomatal and photosynthetic traits related to salinity tolerance in barley, while with a lower LOD score (2.5 < LOD < 3.0), the authors added 11 tentative QTLs for 11 traits to this list. Similarly, Wójcik-Jagła et al. (2013), in two barley mapping populations identified in total 33 QTLs (LOD > 2.5) for physiological parameters measured under short-time drought, but only 11 QTLs were detected with a LOD score >3.0. In the present study, with the aim to search for positional candidate genes associated with QTL confidence intervals, we decided to consider only significant QTLs with a high LOD score.

The QTLs detected in our study are characterized by a large phenotypic effect and a high accuracy of mapping (Mir et al., 2012; Kumar et al., 2017). Assuming that the proportion of phenotype variation explained by a major QTL should be above 10% (Kumar et al., 2017), as much as 95% of the QTLs identified in our study can be considered as the major ones, including all 32 QTLs detected under drought stress and for DSI. Similarly, the 11 significant QTLs detected by Liu et al. (2017), mentioned above, can be classified as the major QTLs, as each of them explained at least 11.2% of variation. On the contrary, in the work of Wójcik-Jagła et al. (2013) only 4 out of 33 QTLs detected in two mapping populations could be considered as the major ones using the above criterion. Moreover, the majority of the QTLs (92.2%) of our study were precisely positioned on the linkage map within confidence intervals below 6.0 cM. It is not a common situation as usually the linkage analysis locates QTLs within the confidence intervals of 10–30 cM (Chander et al., 2008; Li et al., 2008; Capelle et al., 2010; Chen et al., 2010; Kumar et al., 2017). However, in accordance with our results, Bertholdsson et al. (2015) showed a group of 5 QTLs including 3 major QTLs for quantum yield of electron transport of PSII under waterlogging in barley, and all of them but one, were mapped within confidence intervals below 5.0 cM.

### Co-locations of present and known QTL regions

Over the last two decades, QTL for a wide range of traits related to the drought tolerance, including physiological/biochemical characteristics, have been mapped to all seven barley chromosomes (Mir et al., 2012). A precise comparison among these results is not possible owing to the differences in used plant materials and maps, various traits analyzed and diverse methodology applied, however, some interesting observation can be made in regard to the results of the present study. The QTLs identified in our study were mapped to all barley chromosomes, except 4H, with the highest number of QTLs located on chromosomes 2H, 3H and 5H. Based on the overlapping confidence intervals, 11 hotspots were identified that contained together over 60% of mapped QTLs for all the traits, most on 2H (4) and 3H (3). In the previous studies with the same mapping population, aimed at the detection of QTLs underlying yield-related agronomic traits under drought and control conditions (Mikołajczak et al., 2016, 2017; Ogrodowicz et al., 2017), the important role of the regions on chromosomes 2H and 3H was also reported. Moreover, the most significant QTL hotspot on 2H, clustering QTLs for the length of main stem and yield traits (Mikołajczak et al., 2017), as well as heading date (Ogrodowicz et al., 2017), co-localized with a hotspot region (qHS2.1) of the present study. The other region of overlapping QTL hotspots between these studies was found on chromosome 5H. Similarly, Mora et al. (2016) revealed the highest number of QTLs for drought-related morphological and physiological traits on chromosomes 2H and 3H, using a distinct barley gene-pool and environmental conditions. Recently, meta-QTL analysis approach has been developed which is aimed in the integration of data from multiple QTLs studies and has a greater statistical power for the detection of, so called, meta-QTLs (MQTL), and more precise estimation of their genetic effects (Wu and Hu, 2012). Zhang et al. (2017) performed a meta-QTL analysis of drought tolerance in barley using 72 major QTLs described in several studies, and most of QTLs were located on chromosomes 2H, 3H, 5H, and 7H. As a result, MQTLs, integrating QTLs for barley drought tolerance, have been positioned on chromosomes, with some particularly important regions, common to drought and salinity tolerance on 2H (2), 3H (1), and 5H (1).

In the present study no overlapping QTLs identified for a given trait under different water regimes were found. A similar situation was observed by Wang et al. (2014) in QTL mapping for yield-related traits in barley in six diverse environments. Mora et al. (2016) also showed that 80% of the identified QTLs for analyzed traits were specific for a particular environment. According to this observation, we used only the QTLs detected under drought stress conditions and QTLs for adequate DSI, to be subjected for the co-location analysis, projection to barley genome sequence and positional CGs identification.

For the plant water status related traits, a total of 5 QTLs were detected under drought stress conditions and for adequate DSI in our study. Among them, two neighboring regions from chromosome 5H have been pointed in previous studies: QdsiWC.5H_3 for the water content DSI and QdWL.5H_3 for water loss under drought stress co-located with QTL region for RWC determined by Teulat et al. (2001a, 2003), in the close vicinity of *dhn1* and *dhn9* genes encoding dehydrins. This co-location is further confirmed as QdsiWC.5H_3 (*R*^2^ = 23.6%) was mapped into the position of X7136.1 gene, a functional CG which encodes a dehydrin protein. This gene was chosen for mapping based on the transcriptome analysis of parental genotypes of MCam population under drought stress.

For the photosynthetic efficiency category 13 QTLs were detected under drought stress conditions and for adequate DSI. The analysis revealed that two QTL regions from the distal part of a long arm of chromosome 2H: QdABS/RC.2H.2 for PSII light absorption flux per RC and QdET_0_/RC.2H (from hotspot qHS2.4) for electron transport flux in PSII per RC co-localize with two hotspots for chlorophyll *a* fluorescence parameters detected by Guo et al. (2008). These two chromosomal regions were also identified by Wójcik-Jagła et al. (2013) with QTLs for photochemical quantum yield of PSII (Φ_PSII_) DSI, and for photochemical quenching (qP). The region of QdET_0_/RC.2H seems to be especially significant as it explained the highest proportion of the variation (20.5%) and was mapped in the position of AK353596.1 gene (MLOC_65477), a functional CG used for map construction. This gene encodes for an enzyme from the peroxidase class engaged in reactive oxygen species (ROS) detoxification. Different ROS are excessively formatted under drought stress, but they are also produced during the light phase of photosynthesis where about 10% of the pool of electrons is transferred to oxygen forming superoxide anion radical (Foyer and Noctor, 2000). The AK353596.1 gene was selected for mapping based on its differential expression between parental genotypes of MCam under drought (Janiak et al., 2018). Thus, the relationship between this gene and the identified QTL seems to be well-supported. Additionally, two other QTLs related to photosynthetic efficiency of PSII showed the co-locations with QTLs detected previously. The region of Qd(1-B)av.7H_2 from 7H co-located with the QTL for initial fluorescence (F_0_) from the study of Guo et al. (2008) and QdsiDI_0_/RC.5H_2 from 5H overlapped with the chromosomal region of four QTLs for DSI for PSII photosynthetic efficiency and water content (Wójcik-Jagła et al., 2013).

In the osmoprotectant and hormone content category, altogether 11 drought response-specific QTLs were detected. It should be underscored that our study for the first time identified the QTLs for glucose, maltose, sucrose and ethylene content under drought stress conditions in barley. Among them, the QTL from the short arm of 2H, QdsiGlu.2H, for glucose content DSI, overlapped with three other QTLs for WC, WL, and TR_0_/RC (hotspot qHS2.1, around SNP marker 5880-2547) and was mapped near the position of another functional CG, AK356764 (MLOC_21709, an ortholog of Arabidopsis AT3G60750), encoding transketolase. This enzyme is involved in the Calvin-Benson cycle during the light-independent phase of photosynthesis, and according to GO terms, might be also involved in salinity tolerance, gluconeogenesis and water transport. The QdsiGlu.2H region showed the co-location with QTL for WSC concentration at full turgor under drought stress (WSC_100_) designated by Diab et al. (2004). The most interesting result was obtained in the case of six, partially overlapping QTLs for proline, maltose and sucrose content, located in the distal part of a long arm of chromosome 3H. These QTLs explained the highest amount of phenotype variation (54.6–85.9%), were divided among three neighboring hotspots (qHS3.1, qHS3.2, qHS3.3), and coincided with the QTL regions for proline content under drought and salinity stresses identified by Sayed et al. (2012) and Fan et al. (2015), respectively.

In the activity and accumulation of antioxidants category, 3 QTLs were detected under drought stress conditions and for adequate DSI. Here again, for the first time our study determined the QTLs for α-tocopherol (QdATf.6H on 6H) and γ-tocotrienol (QdsiGTt.3H_2) content under drought stress in barley. The region QdsiGTt.3H_2 for γ-tocotrienol content DSI explained 82.1% of phenotype variation for this trait and was included into the hotspot qHS3.2 together with QTLs for proline and sucrose content (co-locations presented above). A third QTL region, QdsiGTf.7H_3 for γ-tocopherol content DSI was mapped on chromosome 7H near SNP marker 6628-1302 and showed the co-location with the QTL for γ-tocopherol content under drought stress detected recently by Templer et al. (2017).

### Prioritization of positional CGs related to drought response-specific QTLs

Although the sequence assembly of the barley genome has been accessible since 2012 (Mayer et al., 2012), only a few studies demonstrated the genetic and physical map integration resulting in numerous putative CG identification (Fan et al., 2015; Piasecka et al., 2017; Templer et al., 2017; Zhang et al., 2017). In the present work, the QTL projection on the genome was extended by the GO enrichment analysis. This allowed an efficient selection of CGs on the basis of their relevance to biological processes related to analyzed trait categories. To the best of our knowledge, it was the first attempt to apply the Gene Ontology-based prioritization to barley QTL analysis. The resulting reduction in the number of genes was almost eight-fold: out of the 1,101 CGs corresponding to the drought response-specific QTLs, we selected 143 significant candidates predicted to be responsible for the variation in the considered traits. Similar findings have been reported by Bargsten et al. (2014) who prioritized rice QTL data leading to the ten-fold reduction in the number of candidate genes. The GO enrichment was also successfully applied to select CGs participating in the genetic determination of the grapevine cluster architecture (Correa et al., 2014), as well as the productivity, growth, and water-use efficiency in poplar (Monclus et al., 2012).

Unquestionably, the thorough examination of CGs revealed in the present study is necessary to confirm their exact role in drought stress response in barley. However, we want to point out that our findings have been supported by results of the global gene expression profiling of parental genotypes that had been used for QTL mapping in our study (Janiak et al., 2018). The differentiated expression due to drought stress was confirmed for 34 CGs from the set of 143 genes selected based on prioritization approach. In the following paragraphs, we characterize the best candidates for further experimental validation of their role in driving drought response. The genes have been carefully chosen based on one of the following criteria: (1) differentiated expression under drought stress, (2) location within overlapping QTL confidence intervals (hotspots), (3) well-documented engagement of their orthologs in model species in drought stress response.

The majority of CGs identified within drought response-specific QTLs for plant water status corresponded to the oxidation-reduction process. It is well-recognized that accumulation of reactive oxygen species (ROS) in the chloroplasts and mitochondria is an early response of plants to decreased water potential inside the cells during adverse stress conditions (Xiong et al., 2002; Choudhury et al., 2013). Many studies have revealed the ROS-mediated retrograde signaling pathway from chloroplasts to nucleus, which results in substantial changes in the expression of the nuclear genes that maintain chloroplasts function and other aspects of plant adaptation to environmental cues (reviewed in Chi et al., 2015). In contrast, an attention has been drawn to the role of ROS as major drivers of cellular oxidative damages (Noctor et al., 2014). Among the barley genes that correlated with the oxidation-reduction process, we highlighted MLOC_69302 encoding ascorbate peroxidase (APX) as a key player involved in the hydrogen peroxide removal. It was reported that *APX* loss-of-function Arabidopsis mutants accumulated more hydrogen peroxide and were significantly more sensitive to different abiotic stresses than the wild type (Koussevitzky et al., 2008). Another gene identified in our study that may be important for barley drought response, MLOC_4579, encodes a flavonol synthase. Flavonols are members of secondary metabolites highly accumulated under various stresses which are involved in ROS scavenging leading to the enhancement of oxidative tolerance (Nakabayashi et al., 2014). Therefore, it can be suspected that the identified barley redox-linked genes contribute to regulation of ROS homeostasis under water deprivation.

The carboxylic acid biosynthesis was the second over-represented process among CGs identified in intervals of QTLs for plant water status. Within this group, MLOC_18300 encoding a member of 9-cis-epoxycarotenoid dioxygenase family (NCED), which catalyzes an essential regulatory step in ABA biosynthesis, was found (Seo and Koshiba, 2002). Drought stress-stimulated accumulation of ABA modulates root hydraulic conductivity and regulates shoot vs. root growth, as well as alters guard cell ion transport, promoting stomatal closure and reducing water loss by transpiration (Roychoudhury et al., 2013; Sah et al., 2016). Another identified gene, MLOC_53947, encodes a putative plastid pyruvate dehydrogenase which contributes to transforming pyruvate to Acetyl-CoA, the precursor for fatty acid biosynthesis (Johnston et al., 1997; Mentzen et al., 2008). It was shown that prolonged drought exerts phospholipid bilayer destabilization and increases its permeability leading to the ion leakage (Bajji et al., 2002; Fang and Xiong, 2015). Therefore, an enhanced biosynthesis of fatty acids may be a crucial strategy of protecting plasma membrane integrity against the damage under drought stress, maintaining osmotic homeostasis and cell turgor pressure, as well as controlling transcellular water and ion transport.

Our analysis clearly showed a putative involvement of numerous genes encoding the heat shock proteins (HSP) in the regulation of photosynthetic efficiency under drought stress in barley. Members of different HSP families perform the molecular chaperone function by participation in the proper protein folding and assembly. Furthermore, they enable disposal of non-functional and aggregated stress-labile proteins (Santhanagopalan et al., 2015). It is well-recognized that HSP proteins play a pivotal role in the protection of photosynthetic components against stress injuries. Recent studies have revealed that stress-induced impairment of photosynthesis results in the reduction of carbon fixation and oxygen evolution, as well as disruption of the linear electron flow. One of the main effects of photoinhibition is the exposure of chloroplasts to excess excitation energy. This increases the generation of ROS by the incomplete reduction of molecular oxygen, which in turn induce remarkable damages in chloroplast proteins, lipids, and pigments (Filek et al., 2015; Pospisil, 2016). The components of photosynthetic apparatus particularly sensitive to oxidative stress are: PSII with its oxygen-evolving complex, the carbon assimilation process driven by RuBisCO, and the ATP generating system (Allakhverdiev et al., 2008). Among genes for HSP identified in this work, the most represented group was orthologs of Arabidopsis AT5G51440 encoding HSP20-like chaperone (MLOC_568 and MLOC_31567). HSP20 proteins are encoded by nuclear multigenic families and are located in different cellular compartments (Lopes-Caitar et al., 2013). In Arabidopsis, 19 genes encoding HSP20 were identified, whereas 36 and 23 *HSP20* genes were described in poplar and rice, respectively (Scharf et al., 2001; Waters et al., 2008; Sarkar et al., 2009). An intriguing over-represented biological process among CGs for photosynthetic efficiency under drought stress was the sulfate assimilation, represented by MLOC_19075, an ortholog of Arabidopsis gene AT4G14680 encoding ATP sulfurylase. Many studies indicate the importance of sulfur as the component of a wide range of compounds with fundamental biological functions, including tolerance to various abiotic stresses (Prioretti et al., 2014). ATP sulfurylases catalyze the first step in the sulfate assimilation pathway. They activate sulfate (${\text{so}}_{4}^{2-}$; a metabolically inert form of sulfur taken up by roots), yielding a high-energy adenosine-5′-phosphosulfate (APS) that is reduced to sulfide (S^2−^) and incorporated into cysteine. In turn, cysteine acts as a donor of highly reactive thiol group (-SH) for numerous S-compounds (Prioretti et al., 2014; Anjum et al., 2015). One of them is glutathione, whose contribution to drought stress tolerance by ROS detoxification and stress-induced signal transduction has been extensively evidenced (Munemasa et al., 2013; Pyngrope et al., 2013; Nahar et al., 2015).

Other relevant biological processes related to the photosynthetic efficiency, over-represented among CGs analyzed in this work, were: short day photoperiodism and red or far red light signaling pathway. Within this group, we highlighted two barley genes, MLOC_6879 and MLOC_53845, orthologs of Arabidopsis *VERNALIZATION INSENSITIVE 3-LIKE 1* (*VIN3-LIKE1*) and *PHYTOCHROME AND FLOWERING TIME 1* (*PFT1*), respectively, involved in the control of flowering time. Many studies have reported a limiting effect of drought stress on plant yield, including early arrest of floral development leading to the interruption of plant reproduction. One of the mechanisms to cope with the water deficit adopted by plants is an acceleration of the flowering process to shorten plant life cycle *via* drought escape. In Arabidopsis, the *FLOWERING LOCUS T* (*FT*) is a master regulator of flowering promotion. It activates the expression of meristem identity genes which control the reprogramming of shoot apical meristem to form flowers (Turck et al., 2008; Kazan and Lyons, 2016). *FT* gene is up-regulated by a transcription factor CONSTANS (CO) and repressed by the product of *FLOWERING LOCUS C* (*FC*). Furthermore, *PFT1* gene was shown to activate expression of *FT* through CO- dependent and independent manner. In contrary, *FC* is epigenetically down-regulated by the vernalization response genes (e.g., *VIN3-LIKE1*; Inigo et al., 2012; Kazan and Lyons, 2016). Taking these data into account, it could be suggested that drought stress occurring at the seedling stage may trigger the up-regulation of MLOC_6879 and MLOC_53845 genes to switch on a drought escape adaptive mechanism by promotion of flowering. This suggestion is supported by a distinctly early flowering of one of the parental genotypes used for QTL mapping, the Syrian line CamB (Ogrodowicz et al., 2017).

We identified several CGs for accumulation of osmoprotectants and hormones within the hotspot regions: qHS2.1, qHS2.3, qHS3.1, and qHS3.2, which suggests their engagement in the complex metabolic networks, probably common to different drought-responsive compounds. As an example, MLOC_174 [a CRT (chloroquine-resistance transporter)-like transporter 3, involved in modified amino acid and glutathione transport], MLOC_40292 (glycerol-3-phosphatase), and MLOC_20354 (gamma-secretase subunit, involved in Notch signaling pathway) were candidate genes for the proline, sucrose, and maltose accumulation. Furthermore, a special attention was given to five genes (MLOC_58506, MLOC_58508, MLOC_58507, MLOC_65368, MLOC_65978) encoding SAUR-like auxin-responsive proteins. Many findings suggest participation of auxin in the plant response to stresses, including drought (Du et al., 2013; Krishnamurthy and Rathinasabapathi, 2013; Rahman, 2013; Shi et al., 2014). It is well-documented, that small auxin up-regulated RNAs (SAUR) are the most numerous family among early auxin response genes, found to be tandemly duplicated in plant genomes (Wu et al., 2012). Accordingly, the SAUR genes identified in this work are clustered within single QTL chromosomal region for maltose content on chromosome 5H. Despite of progressive identification of SAUR genes in different plant species, the functions of most of them, especially in the stress response, remained elusive due to their large genetic redundancy (Ren and Gray, 2015). However, recent studies revealed the role of SAUR genes in hypocotyl elongation and leaf senescence in Arabidopsis, bacterial blight resistance in rice, and drought tolerance in wheat (Chae et al., 2012; Kai et al., 2013; Aoki et al., 2016; Guo et al., 2018). Therefore, it can be hypothesized that observed decrease in maltose content under drought stress conditions, may be regulated by auxin-mediated SAUR genes, but this requires further verification.

The most striking result of GO enrichment was the identification of two genes: MLOC_65646 (encoding molybdopterin biosynthesis protein) and MLOC_22343 (encoding molybdate transporter) involved in the formation of molybdenum cofactor (Moco), which acts as an active compound at the catalytic center of a wide range of oxidation-reduction enzymes (Mendel, 2013). One of these enzymes is aldehyde oxidase which converts abscisic aldehyde to ABA in the last step of the ABA biosynthesis pathway. It was shown that aldehyde oxidase requires the sulfurylated form of Moco for providing its activity (Mendel and Haensch, 2002). This reaction is catalyzed by Moco sulfurase encoded in Arabidopsis by *LOS5/ABI3* gene (Xiong et al., 2001). The study of Li Y. et al. (2013) have revealed that soybean transgenic lines with overexpression of *LOS5/ABI3* showed a reduced electrolyte leakage, enhanced accumulation of proline and antioxidant enzymes, as well as significant increase in ABA content and drought tolerance. The identification on the aforementioned barley genes in the QTL hotspot qHS2.3 for ABS/RC and proline content suggests an interplay between the decreasing photosynthetic efficiency and expression of genes engaged in molybdenum cofactor synthesis, which results in ABA-mediated proline accumulation.

Considering CGs related to the activity and accumulation of antioxidants, we distinguished two genes, MLOC_57100 and MLOC_73233, orthologs of Arabidopsis AT5G54160 (*AtOMT1*), encoding O-methyltransferase. Enzymes belonging to the OMT family catalyze transfer of methyl group to the oxygen atom of the wide range of low molecular weight organic compounds, including flavonoids, alkaloids, and phenylpropanoids. Their methylated products are involved in lignin biosynthesis and response to environmental cues (Lam et al., 2007). Furthermore, it has already been shown that drought stress increased *OMT* transcription in maize, soybean, and grape (Yamaguchi et al., 2010; Liu et al., 2013; Giordano et al., 2016). Since MLOC_73233 was located in the hotspot qHS3.2 that includes QTLs for γ-tocotrienol, proline and sucrose content, it can be hypothesized that O-methylation of intermediate products in the metabolic pathways of these drought-response compounds plays an important role in improving barley tolerance to water deficit.

A putative candidate gene for α-tocopherol accumulation is MLOC_63263 encoding an ortholog of Arabidopsis AT3G47450 (*NITRIC OXIDE SYNTHASE1*; *AtNOS1*). Nitric oxide (NO) is a short-lived, highly membrane permeable, inorganic free radical. It is a crucial signaling molecule mediating diverse physiological processes and defense mechanisms in plants, likewise ROS (Siddiqui et al., 2011). The increased accumulation of NO was reported in wheat and pea under osmotic stress, in parsley under water deficit conditions, and in tobacco under various types of abiotic stimuli (Gould et al., 2003; Kolbert et al., 2005). It is worth noting that NO participates in the ABA-mediated control of stomatal aperture by negative feedback regulation (Wang et al., 2015). The recent study of Yang et al. (2015) showed an interplay between NO and ROS homeostasis. They demonstrated that NO positively regulates the activity of Arabidopsis ascorbate peroxidase APX1 by S-nitrosylation of cysteine residue, which increases its enzymatic activity of hydrogen peroxide detoxification.

## Conclusion

To dissect complex drought tolerance traits and decipher genes underlying resistance mechanisms in barley we employed multidisciplinary approach integrating the latest achievements and tools of physiology, genetics and genomics. Our study proved that thorough physiological/biochemical analysis merged with precisely performed QTL mapping, followed by the use of prioritization tools to dissect genes within physical QTL confidence intervals, is an effective approach to designate the most relevant CGs underlying quantitatively inherited traits. The list of CGs selected on the basis of GO prioritization can be further narrowed down by identifying those that are differentially expressed under drought stress, located within overlapping QTL confidence intervals (hotspots), or have orthologs in model species with well-documented engagement in drought stress response. However, further detailed experimentation using mutants and/or overexpression lines is required to confirm the individual CG relation to the studied trait, before breeding application.

## Author contributions

IS and PK conceived and designed the study; MF, JB-K, and JK designed and coordinated drought stress experiment, contributed to the analysis of the physiological data; KZ and AO performed drought stress experiment; MD, JK, BJ, KH, and KZ generated and elaborated data on physiological and biochemical traits; JG-W and IS coordinated genetic part of the study; KG, JG-W, and DG identified SSRs for mapping; AJ, KG, and JG-W performed selection of functional CGs for mapping; KG, JG-W, JS, and WU genotyped two RIL populations; KG analyzed all genotyping data and constructed two RILs maps and the consensus function map; AK, KM, PO, KK, and PK performed LCam population genotyping and constructed LCam genetic map; KG performed QTL mapping, integration QTLs to physical map, GO enrichment analysis, and together with IS and JG-W performed analysis and interpretation of data; KG, JG-W, and IS wrote the manuscript; All authors commented on the manuscript.

### Conflict of interest statement

The authors declare that the research was conducted in the absence of any commercial or financial relationships that could be construed as a potential conflict of interest.
